# Supplementary material for: Anisotropic nanocrystal superlattices overcoming intrinsic light outcoupling efficiency limit in perovskite quantum dot light-emitting diodes
Source: Nat Commun. 2022 Apr 19;13:2106. doi: 10.1038/s41467-022-29812-5 (PMC9018755; doi:10.1038/s41467-022-29812-5)
Supplement: Supplementary file 1 — Supplementary Information [file 41467_2022_29812_MOESM1_ESM.pdf]

Supplementary Information

Anisotropic Nanocrystal Superlattices Overcoming Intrinsic Light Outcoupling Efficiency Limit in Perovskite Quantum Dot Light-Emitting Diodes

Sudhir Kumar[1†], Tommaso Marcato[1†], Frank Krumeich[2], Yen-Ting Li[3,4], Yu-Cheng Chiu[3], and Chih-Jen Shih[1*]

[1]Institute for Chemical and Bioengineering, ETH Zürich, 8093 Zürich, Switzerland

[2]Laboratory of Inorganic Chemistry, ETH Zürich, 8093 Zürich, Switzerland

[3]Department of Chemical Engineering, National Taiwan University of Science and Technology, Taipei 10607, Taiwan.

[4]National Synchrotron Radiation Research Center, Hsinchu 30076, Taiwan.

[†]Authors contributed equally.

*All to whom all correspondence should be addressed. Email: chih-jen.shih@chem.ethz.ch

Table of Content

1. Theoratical analysis **Error! Bookmark not defined.**

1.1 Perovskite band structure 2

1.2 Anisotropic dielectric confinement 4

1.3 Radiative rate in semiconductor nanocrystals 6

1.4 Role of quantum confinement and exciton fine structure 7

1.5 Role of ANC orientation in thin films 7

2. Characteristics of lead halide perovskite ANCs 8

2.1 Photophysical characteristics and film surface morphology 8

2.2 Optical characteristics and simulations 9

2.3 Role of photon recycling 11

2.4 Internal quantum efficiency (η_{int}) 13

2.5 Birefringence and surface roughness 14

2.6 Role of external electric field 15

2.7 Charge-carrier mobility of ANCs film 16

2.8. Effect of charge balance and recombination 18

3. EL characteristics 18
Supplementary Tables 20
Supplementary Figures 22
Supplementary References 51

1. Theoretical analysis

1.1 Perovskite band structure

In a first approximation, lead trihalide perovskite nanocrystals are modelled as cubic semiconductors with the octahedral symmetry (the O_h point group), where the bandgap occurs at the R point of the Brillouin zone, isomorphic to the Γ point. The six fold conduction and twofold valence bands are contributed from the Pb 6p and halogen 4p orbitals, such that the former possesses p-like (Γ_4^-) symmetry, and the latter s-like (Γ_1^+) symmetry[1].

The symmetry of Bloch states dictates the polarization selection rules for spontaneous emission. We consider as the initial state of the system possessing an electron in the excited conduction band state u_{ck} with momentum k and the electromagnetic field in its vacuum state, i.e. $|u_{ck}; 0\rangle$. The final state has the electron relaxed in the valence band upon emission of a photon of mode j, i.e.$|u_{vk'}\,;\, 1_j\rangle$. The probability for the emission of photons of polarization **e** per unit time and per unit solid angle Ω in the direction (θ, ϕ) is given by the Fermi's golden rule[2]:

$$\frac{d\Gamma}{d\Omega}(\theta,\phi) = \frac{2\pi}{\hbar}\,|\langle u_{vk'}\,;\, 1_j\,|\,\hat{H}_I|u_{ck};\,0\rangle|^2\rho(\theta,\phi,E_g\ =\ \hbar\omega_j) \tag{1}$$

where $\hat{H}_I = -\frac{e}{m}\sqrt{\frac{\hbar}{2\epsilon\omega L^3}}(\hat{p}\cdot e)(\hat{a}_j + \hat{a}_j^\dagger))$ is the field-matter interaction Hamiltonian where ω_j is angular frequency of the emitted photon L^3 is the field quantization volume, $\hat{p}$ is the momentum operator and $\hat{a}_j, \hat{a}_j$ are respectively the electromagnetic field annihilation and creation operators. $\rho(\theta,\phi,E_g\ =\ \hbar\omega_j)$ represents the density of one-photon states of energy equal to the bandgap energy E_g propagating in the direction (θ, ϕ). Accordingly, the polarization of the emitted photons together with the angular radiation pattern are determined by the matrix elements of the operator $\hat{p}$.e. In the following derivations notation from atomic orbitals will be borrowed to label the Bloch states in p-like and s-like bands. As an example |Z⟩ will denote the Block state in the p-like conduction band of orbital angular momentum (l = 1, m_l = 0) given that its spatial symmetry can be described by the same spherical harmonic basis function ($Y_1^0\ (\theta,\phi)$) as a p_z atomic orbital. Similarly, |X⟩ and |Y⟩ denote the remaining states in the conduction band and |S⟩ the valence band states. If we then apply Eq. 1 to a transition between the initial state |Z⟩ and a final state |S⟩ and considering that $\langle S|\hat{p}_z|X\rangle = \langle S|\hat{p}_z|Y\rangle = 0$ by symmetry[2]:

$$\frac{d\Gamma}{d\Omega}(\theta,\phi) = \frac{e^2\omega}{8\pi^2\epsilon_0 m_e^2 \hbar c^3}\,|\langle S|\hat{p}_z|Z\rangle|^2 sin^2\,\theta \tag{2}$$

from which we see that the radiation pattern is the same as that of a classical electric dipole oscillating in the *z* direction. Similarly it can be shown that spontaneous emission from p_x and p_y orbitals is the same of an oscillating electric dipole rotating in the *xy* plane, i.e. circularly polarized light $\sigma^{\pm}$ in the *z* direction.

Next, the spin-orbit interaction leads to the splitting of the conduction band states into a fourfold Γ_8^- state and a two fold Γ_6^-. On the other hand, the valance band states, which have zero angular momentum, are not affected and transform as the Γ_6^+ representation[1, 3].

Once the spin-orbit interaction is included, the orbital angular momentum is not anymore a good quantum number to label the states so we need to use the total angular momentum and its *z* projection, i.e. $|J, J_z\rangle$. Diagonalizing the Hamiltonian leads to the following Bloch states[4]: for Γ_8^- states (J = 3/2)

$$|3/2, 3/2\rangle = -\frac{1}{\sqrt{2}}(|X\rangle + i|Y\rangle)|\uparrow\rangle \qquad |3/2, -3/2\rangle = \frac{1}{\sqrt{2}}(|X\rangle - i|Y\rangle)|\downarrow\rangle$$

$$|3/2, 1/2\rangle = -\frac{1}{\sqrt{6}}(|X\rangle + i|Y\rangle)|\downarrow\rangle + \sqrt{\frac{2}{3}}|Z\rangle|\uparrow\rangle \qquad |3/2, -1/2\rangle = \frac{1}{\sqrt{6}}(|X\rangle - i|Y\rangle)|\uparrow\rangle + \sqrt{\frac{2}{3}}|Z\rangle|\downarrow\rangle$$

and for Γ_6^- states (*J* = 1/2)

$$|1/2, 1/2\rangle = -\frac{1}{\sqrt{3}}(|Z\rangle|\uparrow\rangle + (|X\rangle + i|Y\rangle)|\downarrow\rangle) \qquad |1/2, -1/2\rangle = \frac{1}{\sqrt{3}}(|Z\rangle|\downarrow\rangle - (|X\rangle - i|Y\rangle)|\uparrow\rangle)$$

A good description of the band edge can be obtained by considering only the Γ_6^+ and Γ_6^- states. We notice the latter contains a linear combination of the |X⟩, |Y ⟩, |Z⟩ Bloch states. Following our earlier discussion about Eq. 2, we expect spontaneous emission from optical transitions at the R point to be isotropic in halide perovskites.

In semiconductor nanocrystals (NCs), the quantum confinement leads to strong excitonic characteristics near the band edge. Excitons are two-body bound states of the conduction band electron and the valance band hole coupled via the Coulomb interaction. The wave function follows $\Psi^{exc} = \chi(r_e, r_h)|\Phi\rangle$, where $\chi(r_e, r_h)$ is the exciton envelope function and $|\Phi\rangle$ is suitable symmetric or asymmetric linear combination of the Bloch states of the original carriers. Our earlier results do not change by the introduction of excited states. The envelope function varies slowly in the unit cell and it can be factored out in the expression of the matrix elements of the momentum operators. Hence, the symmetry of the original bulk Bloch states remain to control the polarization selection rules.

The four Γ_6^+ and Γ_6^- states are thus combined into four exciton states. These are not all degenerate but they're split by electron-hole exchange interaction into an optically passive singlet Γ_1^- exciton and a bright triplet Γ_4^- exciton[1, 3]. Given the composition of the latter in terms of single particle Bloch states

and its symmetry, we still conclude that the spontaneous emission is isotropic in cubic lead halide perovskite NCs.

Accordingly, the perovskite radiation behavior appears to be distinct from the conventional zinc blend (ZB) nanocrystals and nanoplatelets (NPLs), e.g. CdSe NPLs. In these crystals, the symmetry of conduction and valence bands is reversed, p-like for the valence band and s-like for the conduction band, such that the valence band maximum (VBM) is composed of J=3/2 states. At the VBM, |3/2, ±3/2⟩ and |3/2, ±1/2⟩ states, or namely, the heavy-hole (hh) and light-hole (lh) states, respectively, have very different effective masses and thus different quantum confinement energies. In quantum dots and NPLs the band edge exciton is almost exclusively composed of heavy-hole states. These states are linear combinations of |X⟩ and |Y⟩ Bloch states, which form a bright plane and emits anisotropically[5]. In NPLs, where the bright plane coincides with the platelet plane, the emission is anisotropic and directed, because the radiative recombination comes from the in-plane dipoles.

1.2 Anisotropic dielectric confinement

The quantum-mechanical analysis presented earlier, however, did not take into account the effect of dielectric anisotropy, which modulates the electric field experienced by the dipoles generated in individual NCs.

Consider a perovskite NC of dielectric constant ϵ_{NC} which is embedded into a medium of dielectric constant ϵ_m under a uniform electric field **E**. The electric field induces the polarization of the NC, resulting in a local electric field, $E^{loc} = -\nabla_{\phi^{loc}}$ where ϕ^{loc} is the local electric potential. Assuming there are no free carriers residing in the domain considered, the electric potential obeys Laplace equation $\nabla_{\phi}^2 = 0$. A solution can then be explicitly related to the external field and be written as

$$E_i^{loc} = f_i(\tilde{\epsilon}, \alpha)E_i \tag{3}$$

where the subscript $i = x, y, z$ coordinates, $\tilde{\epsilon} = \epsilon_{NC}/\ \epsilon_m$ is the dielectric contrast and α is a geometric anisotropy factor, e.g. the aspect ratio (AR). The solutions for spheroids and square cuboids are discussed as follows.

1.2.1 Spheroids

Consider an ellipsoidal with its semi-axes a, b, and c aligned along the x, y, and z axes, respectively. When $a = b$ (spheroids), the Laplace equation had analytical solutions, which yield.[5, 6, 7, 8, 9]

$$f_i = \frac{1}{1+L_i(\tilde{\epsilon} -1)} \tag{4}$$

where L_i are the depolarization factors

$$L_z = \begin{cases} \frac{1-\zeta^2}{2\zeta^3}\left(ln\frac{1+\zeta}{1-\zeta} - 2\zeta\right), & if\ a = b < c\ (prolate) \\ \frac{1+\zeta^2}{\zeta^3}(\zeta - arctan\ \zeta), & if\ c < a = b\ (oblate) \end{cases} \quad (5)$$

$$L_x = L_y = \frac{1 - L_Z}{2}$$

where $\zeta = \sqrt{|1 - a^2/c^2|} = \sqrt{|1 - AR^2|}$. The local field factors for the spheroids are plotted in Supplementary Figure 1 as a function of AR (solid curves). The dielectric contrast relevant was estimated using the experimental high frequency $\epsilon_\infty = 4.7$[10, 11, 12] for bulk $MAPbBr_3$ and the dielectric constant of oleic acid (2.129)[5] for the alkyl ligand shell.

1.2.2 Cuboids

Consider a cuboidal NC with dimensions of *a*, *b*, and *c* in parallel to *x*, *y*, and *z* directions. When $a = b$ (square cuboids), $f_x = f_y$ and there is only one geometric factor AR = a/c The Laplace equation was solved numerically using the commercial finite element package COMSOL Multiphysics. In each simulation, a square cuboid of given AR was embedded in a uniform dielectric medium and placed between a parallel plate capacitor, which was then biased at 1V to ensure a uniform external electric field. Following eq. 3, the local field factor f_i for a given shape was determined by calculating the local electric field at the center of mass of NC, E_i^{loc}. Accordingly, the calculated $f_{x,y}$ and f_z as a function of AR are shown in Supplementary Figure 1 (empty circles).

Supplementary Figure 1 reveals that when AR ≤ 1, the solutions for spheroids and square cuboids are nearly identical. The deviation becomes more significant at intermediate ARs, between 2 to 10, but 3 converges again for AR > 10. We therefore deduce that for the two geometric limits, AR → 0 (rod) and AR → ∞ (disk), the spheroid solutions are asymptotic to those for square cuboids, which are analytically given by

$$\lim_{AR\to 0} f_{x,y} = \frac{2}{1 + \tilde{\epsilon}}$$

$$\lim_{AR\to 0} f_z = 1 \quad (6)$$

$$\lim_{AR\to\infty} f_{x,y} = 1$$

$$\lim_{AR\to\infty} f_z = \frac{1}{\tilde{\epsilon}} \quad (7)$$

The model, as presented above, describes optical absorption in NCs in the visible range. As a matter of fact, the analysis is valid in the long wavelength approximation, in which the NC characteristic length is significantly smaller than the radiation wavelength $L \ll \frac{\lambda}{2\pi n_m}$. In this approximation electrostatic models holds also in the electrodynamic regime where static fields and permanent dipoles are replaced

by frequency dependent fields and oscillating dipoles. The difference of the local field factors along different spheroid and cuboid axes accounts for the experimental observed anisotropy in light absorption by semiconductor NCs. The local field factors arise also in the solution of the reciprocal problem, which models light emission. Considering an electric dipole of moment **d** placed in the center of mass NC, the electric field experienced outside of the NC would be rescaled, effectively identical to the case of placing a dipole of moment $d_i' = f_i d_i$ in the uniform dielectric medium[5, 13, 14]. Clearly, as shown in Supplementary Figure 1, for square platelets (AR > 1), the electric-field screening in the out-of-plane (OP, *z*) direction becomes increasingly stronger than that in the in-plane (IP, *x-y*) direction. Consequently, the radiation from an OP dipole is considerably suppressed as compared to an IP dipole as it scales as the magnitude of the dipole moment squared. In other words, the effect of dielectric anisotropy would be effectively identical to the change of IP dipole ratio, Θ_{IP}, given by

$$\Theta_{IP} = \frac{d_{IP}^2}{d_{IP}^2 + d_{OP}^2} = \frac{2|f_x|^2}{2|f_x|^2 + |f_z|^2} \quad (8)$$

where $d_{IP,OP}$ are IP and OP dipole moments, respectively. Note that when $f_x = f_z$, $\Theta_{IP} = 2/3$, analogous to the case of random dipole orientation in an uniform dielectric medium[5]. Moreover, following Eq. S6-7, for the two geometric limits of rods and disks, ΘIP are given by

$$\lim_{AR \to 0} \Theta_{IP} = \frac{8}{\tilde{\epsilon}^2 + 2\tilde{\epsilon} + 9} \quad (9)$$

$$\lim_{AR \to \infty} \Theta_{IP} = \frac{2}{2 + \left(\frac{1}{\tilde{\epsilon}}\right)^2}$$

For the dielectric constants considered here ($\epsilon_{NC} = 4.7$ and $\epsilon_m = 2.129$), we find that the effect of dielectric anisotropy can increase Θ_{IP} up to 0.91 for AR → ∞. Despite the isotropic nature of halide perovskite electronic structure, the polarization response of anisotropic nanostructures paves to way to achieve directed emission even in halide perovskite thick NPLs or anisotropic nanocrystals (ANCs).

1.3 Radiative rate in semiconductor nanocrystals

The physical picture presented above is also reflected in the quantum-mechanical behavior of dipoles. The spontaneous emission rate, or the radiative rate, for a given dipole in a semiconductor NC can be expressed as follows[3, 7]:

$$\Gamma = \frac{e^2 \omega}{3\pi\epsilon_0 m_e^2 \hbar c^3} n_m |f|^2 \hat{p}_i^2 f \quad (10)$$

where f is the local field factor, n_m is the refractive index of the medium and $\hat{p}$ if is the transition dipole moment. Accordingly, in a spheroid or a square cuboid, the radiative rates resulting from IP and OP dipoles, Γ_{IP} and Γ_{OP}, are given by

$$\Gamma_{IP} = \frac{e^2\omega}{3\pi\epsilon_0 m_e^2 \hbar c^3} n_m |f_x|^2 \hat{p}_i^2 f \tag{11}$$

$$\Gamma_{OP} = \frac{e^2\omega}{3\pi\epsilon_0 m_e^2 \hbar c^3} n_m |f_z|^2 \hat{p}_i^2 f$$

and since $f_z < f_x$ for AR > 1, it appears that in a square platelet or a oblate spheroid, the radiation of out of plane dipoles is inhibited. Together with our findings in Supplementary Section 1.2-1.3, we point out that although the perovskite quantum-mechanical transition at the R point is ”electronically” isotropic, the effect of dielectric anisotropy at the continuum level could considerably influence the radiation pattern generated from its NC. By engineering the perovskite NC shape and orientation with respect to the substrate, in principle, we anticipate that the light outcoupling efficiency can be enhanced.

1.4 Role of quantum confinement and exciton fine structure

The model, as presented in this text, is expected to be valid for NCs in the weak confinement regime when the characteristic length of the NC is significantly bigger than the bulk Bohr radius. First, we believe the assumption of the isotropic nature of the band edge electronic structure might not hold till the monolayer limit. In a highly anisotropic NC, the confinement creates an effective tetragonal crystal field splitting which together with the short-range and shape-dependent long-range components of the Coulomb exchange interaction, induce a fine splitting of the ground state bright exciton in OP and IP levels[15]. A recent spectroscopic study on $CsPbBr_3$ nanoplatelets confirmed a thickness dependent dark-bright exciton fine splitting of up to 32 meV and predicted a maximum splitting between IP and OP of 16 meV[3]. These phenomena are expected to influence Θ_{IP} in two major ways: (i) by inducing an intrinsic difference in TDM matrix elements (electronic structure) and (ii) by inducing a non-negligible difference in the populations of IP and OP levels even at RT (thermodynamic equilibrium). Both effects would positively synergize with the dielectric confinement for high aspect-ratio NCs to lead to an even higher degree of directed emission in strongly confined nanoplatelets.

Nevertheless, given that the NCs explored in this work are only weakly confined and maintain cubic symmetry, engineering the anisotropic dielectric confinement through NC shape is predicted to be the main strategy to increase Θ_{IP} by combining it with ordered assembly in the thin film processing.

1.5 Role of ANC orientation in thin films

Up to this point the model has validity only for single isolated NCs or in the limit of a film of non-interacting NCs perfectly oriented parallel to the film plane, i.e. a film characterized by a delta function orientational distribution. Accordingly, such calculated Θ_{IP} are overestimated and should be regarded as ideal upper bounds.

In order to highlight the importance of controlling the assembly of the individual NC subunit in thin film formation, we extended the model by introducing orientational disorder. If an ANC is not perfectly

oriented with the film, the system is characterized by two sets of coordinates: the local frame of the NC and the global (fixed) frame of the film substrate. Therefore the spatial orientation of an ANC is defined by the coordinates of the vector normal to the X'Y' local plane (θ, ϕ) (primed coordinates refer to the NC frame). We then transformed the $(f_{x'}, f_{y'}, f_{z'})$ vector from the local frame to the lab frame by performing an appropriate rotation. Having now access to $(f_x, f_y, f_z)(\theta, \phi)$ we can perform a first azimuthal angular averaging $\int_0^{2\pi} d\phi\, f_i(\theta, \phi)$ while the averaging with respect to the elevation angle θ was performed accordingly to an appropriate distribution.

Disorder in thin films of rod-like or disk-like particles can be described by a uniaxial orientation distribution function $f(\theta)$ whose only parameter is the angle θ. The distribution is usually characterized by its second momentum S, where S=0 a film with random orientation distribution and S=-0.5 and S=1 for perfectly in-plane or out-of-plane orientation respectively (further details about orientational distribution and averaging in Supplementary Sec. 2.3). Supplementary Figure 2 shows the dramatic effect that film disorder has on the control of $\Theta_{IP,}$ further stressing the importance of engineering both subunit and supramolecular assembly. Our ANC of AR=2.8 shows experimental values of Θ_{IP} up to 75% and we attribute the discrepancy between the theoretical single NC limit value of 80% to the lack of perfect control over NC orientation upon assembly formation.

2. Characteristics of lead halide perovskite ANCs

2.1 Photophysical characteristics and film surface morphology

Supplementary Figure 3 shows photophysical properties of LHP ANCs having a stoichiometric composition of $FA_{0.5}MA_{0.5}PbBr_3$ passivated with the DA ligand. The absorption (Abs) and PL spectra of spin-coated thin films of LHP "thick" ANCs on glass substrate are plotted as a function of wavelength. The thin-film PL spectrum of DA capped ANCs shows a peak emission at 528±1 nm with a narrow full width half maximum (fwhm) of 22.8 nm, while colloidal dispersion of ANC shows an emission maxima at 528±0.9 nm (fwhm = 22.7 nm) and a η_{PL} of 86.2% at an excitation wavelength (λ_{ext}) of 350 nm. Most interestingly, the η_{PL} of DA capped ANC thin-film reaches almost 100% at λ_{ext} of 350 nm. Upon varying the λ_{ext} between 350 and 500 nm with an interval of 10 nm, the ANCs thin-film on glass substrate shows the η_{PL} of 95±5% over the entire excitation range (Supplementary Figure 7). The η_{PL} of the ANC thin-films on the cross-linking X-F6-TAPC and conventional Poly-TPD layers were also investigated to understand the role of underneath hole transporting layer. On the one hand, the η_{PL}, 95.3%, of ANC thin film remained almost unaffected on the X-F6-TAPC layer. On the other hand, the η_{PL} value, 45%, dramatically suppressed upon spin-casting on top of the Poly-TPD layer. Moreover, the ANC thin-film on the Poly-TPD layer shows notably lower η_{PL} between 350 and 420 nm excitation range due to strong absorption of excitation photons through poly-TPD layer (see Supplementary Figure 7 and 9). Moreover, we also observed a notable drop in the average exciton lifetime (τ_{avg}), 44.2 ns, in the ANC thin-film

spin-casted on the Poly-TPD layer (Supplementary Table 1). Undesirable quenching in the η_{PL} and reduced τ_{avg} of ANC thin-film on the Poly-TPD can be attributed to parasitic emission that induce a non-radiative exciton decay channel (Supplementary Figure 7-8). On the contrary, the τ_{avg} = 50 ns in the ANC thin-film on the X-F6-TAPC remain identical with the thin-film sample on bare glass substrate (51.1 ns) (Supplementary Figure 10). Unlike Poly-TPD layer, the photophysical characteristics of the ANC thin-film remain unaffected on the X-F6-TAPC layer, and further confirm the role of underneath layer on the η_{PL}. We also observed significantly short τ_{avg} of 15.95 ns (t_1 = 10.63 ns and t_2 = 29.25 ns) in the thin-film sample of LHP ANCs capped with OLA. The short lifetime in the OLA capped ANC thin-films is resulted due to dielectric quantum confinement effect and confined diffusion of free carriers.[16, 17] The fluorescence intensity maps were acquired using a Leica TCS SP8 confocal microscope. The photoluminescence from the ANCs thin film sample was excited with a focused 405 nm continuous wave laser diode, collected by an oil immersion objective (63x 1.4NA Oil HC PL APO CS2) and detected by a Leica Hybrid Detector (HyD). Supplementary Figure 13 below presents the scanning PL micrograph of our LHP ANCs thin film deposited on the X-F6-TAPC HTL, taken by a confocal laser scanning microscope, Olympus FluoView 3000. According to the microscope specifications, the image lateral resolution is 120 nm. As shown, we observed uniform PL emission at micrometer scale throughout the ANCs thin-film surface. We have also examined the LHP ANC films deposited on X-F6-TAPC HTL using the scanning electron microscope (images see Supplementary Figure 14), which shows a consistent and smooth surface morphology over a large area.

To understand the recombination mechanism of ANCs thin films on the bare glass and X-F6-TAPC layer, the η_{PL} and the time-resolved PL at different temperatures was investigated (see Supplementary Figure 11 and Figure 12). Supplementary Figure 12a reports the absolute η_{PL} of samples at different excitation power. With increase excitation power, the η_{PL} in the both samples show a slightly drop with growing excitation power upto 120 μW cm^{-2}. Later on, a substantial descent in the absolute η_{PL} at 100% excitation power (1.2 mW cm^{-2}) is attributed to exciton-exciton interactions, and results a fast biexcitonic Auger type non-radiative recombination.[18, 19] To further confirm the role of X-F6-TAPC layer on the recombination mechanism the PL decay curves of ANC thin films were characterized by varying the excitation power (Supplementary Figure 12b). Upon increasing the excitation power, both samples show a noticeable increase in the fast component. Thus, both samples show a steady descent in the average PL lifetime as excitation power increases from 8.5 pJ cm^{-2} to 8.5 nJ cm^{-2}, which is typical behavior of fast biexcitonic recombination that often resulted due to fast Auger type processes.[18] Note that both samples exhibit the absolute η_{PL} of almost 100% at low excitation power, therefore, the dominated fast component is not resulted due to trap assisted recombination of free carriers that often resulted in bulk and NCs with surface defects.[18]

2.2 Optical characteristics and simulations

The refractive indices (n) of hole transporting materials, Poly-TPD and X-F6-TAPC, were estimated through fitting the non-absorbing region, 500-850 nm, of optical index data of X-F6-TAPC and Poly-TPD was fitted with the Sellmeier dispersion model. Moreover, the non-absorbing region, 550-850 nm, of optical index data of LHP ANC thin-film was fitted with the Sellmeier dispersion model. The optical constants, A_1, A_2, A_3, B_1, B_2, and B_3, of all thin films were calculated by fitting the raw data psi (ψ) and delta (Δ) plot with the SENTECH SpectraRay2 (SR2) software (Supplementary Table 2). Furthermore, the analysis was completed when Tauc–Lorentz (TL) model was applied in order to account for optical absorption. A reasonable fit was obtained with two TL oscillators in the range of 500–700 nm. The Poly-TPD and X-F6-TAPC shows the refractive indices of 1.65 and 1.54, respectively, at the PL emission maxima 530 nm of ANCs. We have also chosen a low refractive index electron transporting material, 3TPYMB (n = 1.66 at 528 nm).[20] Moreover, we have carried out additional measurements to characterize the refractive index of the ANCs thin film and confirmed that the refractive index of n = 1.76±0.05 at 530 nm. We attribute the low refractive index to: (i) the relatively long alkylamine ligand, n-decylamine (DA) used in our synthetic protocol, and (ii) excess amount of unreacted nucleating agent, oleic acid (OA), and DA ligands in the colloidal dispersion. In the organic-inorganic hybrid perovskite NC systems, for example, a recent paper suggests a refractive index value of 1.82 at 620 nm in the $MAPb(Br_xI_{1-x})_3$ OIHP NCs, where the excess unreacted ligands were removed using anhydrous methylacetate.[21] Note that iodide mixing intrinsically increases the NC refractive index. On top of all these consideration, a back of the envelope calculation using simple Maxwell-Garnett effective medium theory supports the experimental observed value. In first approximation, we can estimate the volume fraction as given only by the ratio between the bare ANC volume $V = L^2 d$ and that of the enclosed nanocrystal $V^* = \left(L + 2d_{\text{lig.}}\right)^2 (d + 2d_{\text{lig.}})$, resulting in a value of 0.615. The Maxwell-Garnett formula predicts a refractive index of 1.87 for ANC and ligands composite film. Note that the volume fraction of any unreacted ligand in the ANCs thin films have not been considered for the refractive index estimation. Therefore, our experimental findings are analogous to the theoretical predictions.

To gain more understanding, the optical simulations were performed to understand the role of hole transporting layer (HTL) and electron transporting layer thicknesses and their refractive indices on the light out-coupling efficiencies (η_{out}) in the optimal device architecture (Supplementary Figure 15). Supplementary Figure 15a shows the contour plot of the η_{out} of optimal device as a function of HTL, X-F6-TAPC, and ETL, 3TPYMB, thicknesses by using the horizontally oriented (Θ_H) TDM of 1, and optimized ANC emission layer thickness. The optical simulations were performed by assuming the zero electrical loss, while the charge carrier recombination zone was assumed in the middle of the ANC thin-film emission layer. As we have not observed any noteworthy change in the η_{out} by varying the position of recombination zone in the LHP emission layer. Upon varying the layer thicknesses of low refractive index cross-linkable HTL, X-F6-TAPC (n = 1.54 at 528 nm), and ETL, 3TPYMB (n = 1.66 at 528 nm),

between 5 and 200 nm, the η_{out} ranges between 2.5 and 47%. Most interestingly, the optimal LED device with an emission layer having a corresponding exciton TDM either partially, 0.72, or entirely, 1, oriented toward horizontal direction demonstrates a highest η_{out} of 32.4 and 41.15%. Our optical simulation results further affirms the significance of low refractive index carrier transporting materials, X-F6-TAPC as HTL and 3TPYMB as ETL, and horizontal orientation of exciton TDM on the η_{out} (Supplementary Figure 15b-d). Our simulations confirms that the lower and upper η_{out} greatly enhanced from 17 to 29% and 37 to 54%, respectively, as the orientation of TDM increases from 0.67 (isotropic) to 1 (completely horizontal). Particularly, the anisotropic nanocrystal superlattices (ANSLs) film of LHP ANCs show a notably higher η_{out}, 19 - 39%, by replacing the high refractive index HTL and ETL (n = 2.0) with low refractive index (n = 1.4) counterparts (Supplementary Figure 15b).

2.3 Role of photon recycling

Consider an EML deposited on a substrate, the escape probability in a half-space for a given Hertzian dipole oriented with angle θ with respect to the film *z*-axis, P_{out}, is given by[22, 23]:

$$P_{out} = \frac{1}{2} - \frac{1}{2}\sqrt{1-\left(\frac{n_0}{n_1}\right)^2}\left(1+\frac{n_0^2}{2n_1^2}\left(1-\frac{3}{2}\sin^2\theta\right)\right) \tag{12}$$

where n_1 and n_0 are the refractive indices of EML and substrate, respectively. Note that if the dipole is isotropically oriented, Eq. (12) is reduced to a well-known expression, $P_{out}^{isotropic} = \frac{1}{2}\left(1-\sqrt{1-\left(\frac{n_0}{n_1}\right)^2}\right)$. However, Eq. (12) above only considers a single emitter oriented at a particular angle to approximate the extraction probability for the entire film. To properly model the effect of individual dipoles within the EML, the quantities has to be appropriately averaged with respect to the collective orientation distribution function of the dipole ensemble $f(\theta)$, which can be expanded as follows[24]:

$$f(\theta) = \frac{1}{2} + \frac{5}{2}\langle P_2\rangle P_2(\cos\theta) + \frac{9}{2}\langle P_4\rangle P_4(\cos\theta) + \cdots \tag{13}$$

where, $P_L(\cos\theta)$ is the Legendre polynomials of order L and $\langle P_L\rangle$ its orientational average. The latter are also known as the order parameters, and in particular $\langle P_2\rangle = S$, which closely relates to the average ratio of horizontally oriented dipoles, Θ_H, extracted from the angle-dependent PL spectroscopy[25] following:

$$S = \frac{1}{2}\left(2 - 3\Theta_H\right) \tag{14}$$

Taking into account this effect, the average escape probability, $\langle P_{out}\rangle$, follows:

$$\langle P_{out}\rangle = \frac{\int_0^{\pi} d\theta \sin\theta\, f(\theta) P_{out}(\theta)}{\int_0^{\pi} d\theta \sin\theta\, f(\theta)} \tag{15}$$

We can reconstruct the distribution function given its first order orientation parameter employing a maximum entropy approach that yields[24]:

$$f(\theta) = \frac{\exp(a_2 P_2(\cos\theta))}{\int_0^{\pi} d\theta \sin\theta \exp(a_2 P_2(\cos\theta))} \tag{16}$$

where the parameter a_2 has to self-consistently fulfill:

$$S = \frac{\int_0^{\pi} d\theta \sin \quad P_2(\cos\theta)\exp(a_2 P_2(\cos\theta))}{\int_0^{\pi} d\theta \sin \quad \exp(a_2 P_2(\cos\theta))} \tag{17}$$

Next, the effect of photon recycling comes into play. Given the optical density, OD, of the EML at the emission wavelength (in our system OD = 0.011 at 528 nm), the internal photoluminescence quantum yield within the EML, ϕ, taking into consideration both dipole orientation and photon recycling[25, 26], follows:

$$\phi_{ext} = \frac{\phi\eta_{esc}}{1-\phi+\phi\eta_{esc}} \tag{18}$$

where ϕ_{ext} is the external photoluminescence quantum yield determined in air, η_{esc} is the light escaping efficiency given by:

$$\eta_{esc} = 10^{-\frac{OD}{2}}(P_{out}^{air} + P_{out}^{glass} + 10^{-OD}(P_{out}^{glass} - P_{out}^{air})) \tag{19}$$

Note that P_{out}^{air} and P_{out}^{glass} correspond to $\langle P_{out}\rangle$ using n_0 values of 1.0 and 1.51, respectively.

Accordingly, with known EML parameters, n_0, n_1, Θ_H, ϕ_{ext}, and OD, one can calculate $\langle P_{out}\rangle$, ϕ, and η_{esc} by solving Eqs. (12) – (19) numerically. The analysis presented here would allow us to reveal the effects of photon recycling and dipole orientation.

Supplementary Figures 16 shows the calculated orientational distributions $f(\theta)$ (a), the escape probabilities in air P_{out}^{air} (b) and glass P_{out}^{glass} (c), and the dependence of internal and external quantum yields, ϕ_{ext}- ϕ for different EML Θ_H values. In particular, in Supplementary Figure 16 (d), we compare the calculated ϕ_{ext}- ϕ relations with that in Ref. [27] (black curve), and find that the deviation between internal and external quantum yields is greatly reduced. We attribute it to the enhanced escape probability in our thin-film system, resulting from the relatively low refractive index and film thickness of EML. On top of that, the deviation is reduced and eventually diminished when the internal quantum yields approaches unity.

We further look into the effect of dipole orientation Θ_H. Supplementary Figure 16(d) suggests that the calculated ϕ_{ext}- ϕ dependence only weakly varies with Θ_H. In particular, when ϕ_{ext} is nearly unity, which is the case in our systems, the effect of Θ_H becomes negligible.

In summary, the theoretical analysis presented here takes into account the effects of photon recycling and dipole orientation in EML. Based on the calculated ϕ_{ext}- ϕ profiles, we conclude that due to (i) the

low refractive index and small thickness of EML and (ii) the near-unity external photoluminescence quantum yield, photon recycling essentially plays a negligible role in our NC solids, similar to the guest-host EML thin films in OLEDs.

2.4 Internal quantum efficiency (η_{int})

It is well known that the radiative rate and η_{PL} of an emitter are dependent on the inhomogeneous environment surrounding it.[28] Therefore, an accurate estimation of the internal quantum efficiency in a LED device requires the extraction of the internal photoluminescence quantum yield at the specific position of the emission zone. The internal PLQY values can differ from those reported in the previous section (Role of photon recycling), due to the effects of, for example, the self-interaction with the reflected dipole radiation at the cathode. Given the internal η_{PL} as the starting value, we have calculated η_{int} in actual LED device architecture as a function of Θ_H, using the modified Chance, Prock and Silbey approach[28] as implemented in the software package Setfos, as shown in Supplementary Figure 17.

Supplementary Figure 17 presents the calculated escape probability (a), internal PLQY (b), and the final device internal quantum efficiency η_{int} (c) as a function of Θ_H, for different external PLQY values. Note that our calculations have taken into account: (i) the photon recycling effect, (ii) the actual device thin-film architecture, and (iii) the TDM orientation effect, given a known value of external PLQY. Clearly, as shown in Supplementary Figure 17(c), the calculated internal quantum efficiency η_{int} appears to inherit the weak dependence on dipole orientation from the internal η_{PL} (b), due to the effect of photon recycling, particularly when the external PLQY is high.
Our analysis reveals a complex picture suggesting that not only the outcoupling efficiency, η_{out}, but also the internal quantum efficiency, η_{int}, are affected by Θ_H. The η_{int}-Θ_H dependence is mediated through the photon recycling effect, particularly relevant in moderate η_{PL} emitters of high OD. However, for the near-unity η_{PL} emitters, e.g., our perovskite NC solids, the Θ_H effect on η_{int} is nearly negligible. As such, the external PLQY remains to be the dominant factor determining η_{int}.

With the above discussions in mind, we have estimated the maximum η_{ext} (assuming unity charge balance efficiency) of our LED device. The parameters used in our calculations are as follows: (i) Θ_H = 0.73 (from our angle- and polarization- dependent PL measurement), corresponding to S = -0.097, (ii) the EML refractive index n = 1.76±0.05 (from Ellipsometry), (iii) EML thin-film OD of 0.011 at 528 nm (from the absorption measurement), and (iii) the external η_{PL} of 98% (from the absolute QY measurement).

Accordingly, our calculations yield the total escape probability of 50.4%, the internal η_{PL} of 99%, and the resulting η_{int} of 95%. Together with the calculated η_{out} of 32.2%, we are able to calculate the maximum attainable η_{ext} following:

$$\eta_{ext} = \chi\, \eta_{int}\, \eta_{out} = 1 * 0.95 * 0.322 = 30.55\% \qquad (20)$$

Note that we consider that both singlet and triplet excitons contribute to photon generation due to the strong spin-orbit coupling and low exciton fine splitting energies in LHPs[29, 30].

Furthermore, the previous models showed how photon-recycling effects are a saturated degree of freedom in thin films of high η_{PL} LHP nanocrystals with respect to η_{ext} maximization and hence the control of TDM orientation remains the only viable intrinsic strategy for efficiency boosting in near unity η_{int} systems. Despite the relatively low impact on overall efficiency due to the limitation in aspect ratio control (the estimated maximum η_{ext} for perfect cubes is $\eta_{ext} = 1 * 0.94 * 0.298 = 28\%$), our report represents the first observation and rationalization of the effect of TDM control on EL efficiency in LHP NCs with isotropic band structure.

2.5 Birefringence and surface roughness

Indeed, the optical birefringence is often observed in thin films made by microscopic building blocks resulting from the anisotropy of molecular polarizabilities[31, 32]. For example, uniaxial films containing molecules that are of preferentially horizontal orientation could exhibit negative birefringence, changing the light propagation. In these materials, one can observe a difference between ordinary and extraordinary extinction coefficients, which can be used to quantify the TDM distribution.

However, although our EMLs are essentially comprised of individual nanocrystals having emission polarizability, the collective optical properties of EML characterized from far-field ellipsometry still suggests that the film behaves like a composite material containing low-concentration anisotropic inclusions surrounded by isotropic organic ligand matrix. Based on our results, we suppose that similar to the host-guest EML systems in OLEDs, the spectroscopic ellipsometry is not sufficiently sensitive to quantify the degree of anisotropy for the minority inclusions[33].

The analysis has motivated us to use GIXRD and the angle- and polarization- dependent PL to characterize superlattice and the TDM orientations of our NC solids.

To reveal the effect of surface roughness on our HTL (X-F6-TAPC) and ETL optical properties, we examined a model that is highly sensitive to surface roughness. This model considers a rough dielectric layer placed between air and the layer of interest. In the first approximation, the layer was treated as a composite of air (voids) and thin film material with 50% volume fraction, whose dispersion was estimated using the Bruggemann effective medium approximation[34]. Next, the thickness of the rough layer was then computationally tuned to minimize the root-mean square (RMS) errors for the Ψ and Δ fits.

Supplementary Figures 18(a) - 20(a) show the optimized Ψ and Δ fits together with the RMS for our representative perovskite nanocrystal EML and X-F6-TAPC HTL films. In both systems, the optimization process for the Tauc-Lorentz fit performed by the package SpectraRay gave a thickness of

the rough layer of zero. In all cases, the experimental Ψ and Δ results are nicely fitted, suggesting that the degree of surface roughness is significantly smaller than the wavelength, such that the scattering effect is negligible.

Note that all fittings in Supplementary Figures 18(a) and 20(a) were performed considering isotropic thin films without birefringence. The low RMS in fitting the ellipsometry data suggests that the isotropic model is appropriate to model the optical properties of our EML and HTL.

Nevertheless, in order to further examine the effect of birefringence in our EML, we consider an uniaxial birefringent material having complex refractive indices (n, k) along the ordinary (o) and extraordinary (e) directions, giving in total four dispersions, n_e, n_o, k_e, and k_o. We estimated the maximum differences between o and e directions, $\Delta k = k_o - k_e$ and $\Delta n = n_o - n_e$, using our order parameter S = -0.08 and that reported in organic materials with nearly identical S (CBP S=-0.07[35]), respectively. The calculated Tauc-Lorentz dispersions for n_e, n_o, k_e, and k_o are shown in Supplementary Figure 18(b). We then used the four dispersions in optical simulations in Setfos to calculate the outcoupling efficiency η_{out} in our LED stack. As compared to the isotropic model used in our manuscript, we find that the maximum difference in η_{out} is only 0.5%. With the above analysis in mind, we confidently claim that the effects of birefringence and roughness are negligible in our EML system.

2.6 Role of external electric field

In order to ensure a spatially uniform external field for accurate polarizability extraction, we used a fictitious plane plate capacitor with 1V voltage difference across it, with the thickness and area significantly larger than the nanocrystal placed in the center, following the strategy proposed by Becker et al[3]. Such macroscopic model does not take into account the field dependent effects or optical nonlinearities such as electron/hole correlation effects, thereby giving a field independent macroscopic polarizability. Indeed, it is known that despite current flow, an out-of-plane electric field of up to 3×10^4 kV/m can build up across the emissive layer within an OLED device, so more consideration may be required to take into account the effect.

On the other hand, excitons in quantum confined nanocrystals can possess static and dynamic (TDM) polarizabilities, which are usually very high[36]. In particular, the dynamic polarizability, which controls the field dependence of the radiative rate has been observed to be negative in CdSe nanoplatelets and hybrid perovskites[37, 38], so that radiative recombination is hindered at high electric field strengths as the electron and hole overlap is distorted.

To reveal the effect of electric field strength, we consider IP and OP dipoles residing in a nanocrystal experiencing a vertical external electric field along the OP (z) direction. The effect of electric field strength is mediated through the dynamic polarizability[37], and the TDM in the linear response regime can be approximated as follows:

$$\mu_i' = \mu_i^0 + X\mathbf{E}_\mathbf{i}^{\mathbf{loc}} = \mu_i^0 + X\sum_j f_{ij}\mathrm{E}_j \tag{21}$$

where X is the dynamic polarizability and subscripts i and j refers to coordinates x, y, or z. Therefore, the radiative rates for an IP and OP dipoles within a nanocrystal, Γ_{IP} and Γ_{OP}, under an OP external electric field, E_z, are given by:

$$\Gamma_{IP} = \frac{e^2\omega}{3\pi\epsilon_0 m_e^2\hbar c^3} n_m \left|\mu_{x,y}\right|^2 \approx \frac{e^2\omega}{3\pi\epsilon_0 m_e^2\hbar c^3} n_m \left(\left(\mu_{x,y}^0\right)^2 + 2X\mu_{x,y}^0 f_{xz,yz}\mathrm{E}_z\right)$$

$$\Gamma_{IP} = \frac{e^2\omega}{3\pi\epsilon_0 m_e^2\hbar c^3} n_m \left|f_{xx,yy}\right|^2 (\hat{p}_{if}^2 + 2X\hat{p}_{if} f_{xz,yz}\mathrm{E}_z) \tag{22}$$

$$\Gamma_{OP} = \frac{e^2\omega}{3\pi\epsilon_0 m_e^2\hbar c^3} n_m |\mu_z|^2 \approx \frac{e^2\omega}{3\pi\epsilon_0 m_e^2\hbar c^3} n_m ((\mu_z^0)^2 + 2X\mu_z^0 f_{zz}\mathrm{E}_z)$$

$$\Gamma_{OP} = \frac{e^2\omega}{3\pi\epsilon_0 m_e^2\hbar c^3} n_m f_{zz}^2 (\hat{p}_{if}^2 + 2X\hat{p}_{if} f_{zz} E_z)$$

where quadratic terms in the electric field have been neglected.

By looking into Eq. (22), for an x-oriented IP TDM within a nanocrystal, its response to an electric field parallel to the z direction would depend on the xz component of the NC polarizability, and analogously, an z-oriented OP TDM depends on the zz component. For an IP dipole (top equation in Eq. (22)), because the polarizability of a given nanocrystal is a diagonal tensor, or f_{xz} is zero, one can observe that the effect of terms depending on the electric field E_z become zero. We can therefore safely claim that the radiative rate of an IP dipole would not be affected by the strength of a given vertical electric field. On the other hand, for an OP dipole (bottom equation in Eq. (22)), we observe that a vertical electric field could decrease the OP dipole radiative rate, because f_{zz} is nonzero and the dynamic polarizability X is negative.

In other words, the theoretical analysis presented here suggests that: by increasing the strength of a vertical electric field, the effect of emission polarizability, which is originated from the increase of IP dipole radiative rate and the decrease of OP dipole radiative rate, would be even more amplified. Accordingly, we can conclude that as long as a given NC is horizontally oriented, introducing a stronger vertical electric field would not randomize the TDM orientation, but rather making it more horizontal (or in-plane).

2.7 Charge-carrier mobility of ANCs film

Hole only device (HOD). Patterned ITO coated glass substrates were sequentially sonicated in soap-solution, acetone, and isopropanol each for 20 minutes. The substrates were then exposed to oxygen plasma for 10 min. in diener plasma cleaner using 80% lamp power. Firstly, a 20 nm PEDOT:PSS solution was spin-coated at a spin rate of 6000 rpm for 50 s and annealed on a laminar bench at 120 °C for 30 min. Subsequently, all substrates were transferred into the glovebox. Then, ANCs film was also

spin-casted at 2500 rpm for 40 s. Finally, all substrate transferred into an evaporation chamber to sequential evaporation of a 5 nm molybdenum oxide (MoO_x) and a 100 nm gold (Au) layers in the high vacuum chamber at ~1×10^{-7} mbar.

Electron only device (EOD). Patterned ITO coated glass substrates were sequentially sonicated in soap-solution, acetone, and isopropanol each for 20 minutes. The substrates were then cleaned using oxygen plasma treatment. At the outset, a 20±5 nm SnO_2 layer was spin-casted onto the ITO and annealed on a laminar bench at 150 °C for 75 min. Thereafter, the substrates were transferred to nitrogen filled glove box for spin-coating of the ANCs layer at 2500 rpm for 40 s. The substrates were then transferred in the evaporation chamber. Later on, a 20 nm TPBi, 1 nm LiF, and 70 nm Al layers were the sequentially deposited by thermal evaporation chamber at ~1×10^{-7} mbar.

Characterization of carrier only devices. The *J-V* characteristics of both HOD and EOD devices were characterized using an Agilent B1500A Semiconductor Analyzer Version 202009 and a Probe Station under nitrogen filled glove box.

We fabricated the hole- and electron-transport-only devices to characterize electron and hole mobilities in our EML. The device architecture are as follows: ITO/PEDOT:PSS/EML/MoO_3/Au and ITO/SnO_2/EML/TPBi/LiF/Al, respectively. The *J-V* characteristics would allow us to approximately estimate the carrier mobilities according to Röhr et al.[39] considering the space charge limited current (SCLC) regime (Supplementary Figure 21b and 22b). The extracted region was subsequently fitted with the Mott-Gurney law (Supplementary Figure 21a and 22a):

$$J = \frac{9}{8}\epsilon_0\epsilon_r\mu_0\frac{V^2}{L^3} \tag{23}$$

Where ϵ_r and μ_0 are the material permittivity and mobility respectively and L is the electrode-to-electrode device thickness. As the model does not allow the independent extraction of EML permittivity, the product of $\epsilon_r\mu_0$ was used as the initial guess in the drift-diffusion modelling. The choice of SCLC interval was subsequently determined by maximizing its fitting linearity in the log-log plots.

Next, the model was extended to consider the field dependence of the mobility via a modified Poole-Frenkel model following[40] (Supplementary Figure 21c-d and 22c-d):

$$J = \frac{9}{8}\epsilon_0\epsilon_r\mu_0\frac{V(V+V_\beta)}{L^3}\exp\left(\gamma\sqrt{\frac{V}{L}}\right) \tag{24}$$

Where V_β is a fitting parameter which is fixed at a value which maximizes the linearity of the *J-V* curve on a $\log\frac{J}{V(V+V\beta)}$ - $\sqrt{V/L}$ plot and γ is the parameter that determines the mobility field dependence.

Supplementary Figure 21(d) and 22(d) present the extracted electron and hole mobilities, respectively. As shown the electron mobility is about one order of magnitude lower than that of hole. The hole and electron mobilities in the ANC films are showing similar trend with bulk $MAPbI_3$ films.[41]

2.8. Effect of charge balance and recombination

The recombination profile of injected hole and electron in the optimized device architecture was computed using the Setfos 5.2 drift-diffusion module. The hole and electron mobilities for the X-F6-TAPC/3TPYMB and EML layers were taken from the literature[42, 43] and our hole-only/electron-only devices, respectively (Supplementary Section 2.7 and Supplementary Figure 21 and 2). Supplementary Figure 32 shows the recombination profile as a function of device layer thickness. Upon varying the driving voltage between 2.75V and 6.75V, it is observed that the majority of carriers recombination taking place within the EML rather than the adjacent transport layers. The recombination zone within the EML is broad, with its maximum located at the HTL/EML interface, which gradually increases with the driving voltage. Indeed, as discussed in the main text, the X-F6-TAPC layer effectively blocks the electron transport, and the electron mobility of 3TPYMB ETL is nearly three orders of magnitude higher than the hole mobility of X-F6-TAPC. As a result, a large population of injected electrons reaches and recombines with holes at the HTL/EML interface.

3. EL characteristics

Considerable experimental efforts were made to optimize the auxiliary layers, including hole transporting layers (HTLs), electron transporting layers (ETLs), and electron injection layers (EILs). To assess the role of ETL and EIL on the EL performance, the LED devices consisting the 3TPYMB ETL and Liq EIL were fabricated. Upon replacing the conventional TPBi layer with the 3TPYMB, the device shows the maximum η_{ext} of 10.6% and η_{CE} of 46.6 cd A^{-1}, which are over 80% and 88%, respectively, higher than that of control device (Supplementary Table 3). We attribute the higher efficiencies to low electron injection barrier and low refractive index, n = 1.65, that tenders a high η_{out} through increasing the exciton out-coupling to air mode. Furthermore, an optimal 3TPYMB thickness, 50 nm, possesses an adequate distance from the aluminum cathode, thus effectively suppress the surface plasmon polariton (SPP) losses.[20, 44, 45] The device performance further excelled (η_{ext} = 24.96% and η_{CE} = 103.4 cd A^{-1}) upon introducing an 18±1 nm thick cross-linkable X-F6-TAPC layer between PEDOT:PSS and perovskite emission layer. The superior device performance is attributed to following factors, including, (i) near-unity thin-film η_{PL}, (ii) highest occupied molecular orbital (HOMO) energy level (-5.63 eV) of X-F6-TAPC that reduce the hole injection barrier between PEDOT:PSS and EML thus enables an energy cascade route for hole injection[43, 45], (iii) effective energy barrier to confine the injected carriers, electron and hole, within the EML due to a high LUMO energy level (-1.63 eV) in the X-F6-TAPC and a low-lying HOMO energy level (-6.8 eV) in the 3TPYMB [42, 43, 45], (iv) low refractive indices in the X-F6-TAPC (n = 1.54) HTL, perovskite EML (n = 1.73), and 3TPYMB (n = 1.66) ETL that greatly

enhance the η_{out} (details see Supplementary Section 2)[20, 44, 46], and (v) X-F6-TAPC layer effectively reduces the current leakage. We have also tested two other electron-transporting materials, 2,4,6-tris[3-(diphenylphosphinyl)phenyl]-1,3,5-triazine (PO-T2T) and 4,6-bis(3,5-di(pyridin-3-yl)phenyl)-2-methylpyrimidine (B3PYMPM) in the LED devices to attain better EL performance. The devices with PO-T2T as ETL demonstrate significantly lower efficiencies (η_{ext} = 10.34% and η_{CE} = 42.52 cd m^{-1}) than that of champion device, while higher efficiencies than the device consisting B3PYMPM as ETL (η_{ext} = 8.69% and η_{CE} = 36.11 cd m^{-1}). Particularly, the poor device performance in the devices with the PO-T2T and B3PYMPM ETLs than the 3TPYMB based device because of (i) large electron injection barrier due to high LUMO energy level (-2.8 eV) and relatively higher refractive index of (n = 1.74 at 528 nm) in PO-T2T, while a considerably higher refractive index (n = 1.86 at 528 nm). These factors either significantly reduce the η_{out} by increasing the total internal reflection (TIR) or unfavorably enable a carrier injection imbalance during the device operation.[20, 44] We observed the consistent EL emission maxima at 528 nm in all devices without any parasitic emission from underneath HTL, poly-TPD and X-F6-TAPC (Supplementary Figure 27).

Our champion device exhibits ultra-pure green emission chromaticity with the $CIE_{x,y}$ coordinates ranging between (0.168, 0.771) and (0.174, 0.795), which are almost identical with the standard Rec. 2020 color coordinates (0.170, 0.797). The optimal LED devices demonstrate “greenest” EL emission, to our knowledge, that covers 99.5% and 99.8% of the Rec. 2020 standard gamut area in the CIE 1931 and the CIE 1976 color space, respectively, upon substituting from the standard green chromaticity coordinates.[47, 48]

The optimal device architecture was utilized to test the EL characteristics of isotropic NCs. Supplementary Figure 38 shows the maximum η_{ext} of 12.82% with a maximum η_{CE} and η_{PE} of 53.08 cd A^{-1} and 47.3 lm W^{-1}, respectively. The devices based on the isotropic NCs (INCs) emissive layer show significantly lower performance than that of anisotropic NCs (ANCs) counterparts. These results further affirm that the horizontal orientation of exciton TDM plays a crucial role in the high η_{ext} of champion device.

As shown in Supplementary Figure 40, our optimal device exhibits a 0.73V rise in the operational voltage (ΔV), when the device stressed at a constant current density of 0.5 mA cm^{-2}. Moreover, the ΔV value in the control device also increases to 0.51V during the operational stability measurement. Generally, the increase in driving voltage is occurred to maintain the initial current density throughout the operation. The change in voltage during operational stability could be attributed to formation of resistive paths due to ion migration and perovskite emissive layer degradation under electric field. We observed tremendous spectral stability, as peak EL spectra of electrically stressed devices remain consistent with the fresh LED counterparts (Supplementary Figure 41). We attribute the improved operational lifetimes in the X-F6-TAPC based devices to the following factors: (i) the cross-linked X-

F6-TAPC layer prevents a direct contact between PEDOT:PSS and EML, thereby minimizing the non-radiative quenching, (ii) the X-F6-TAPC layer has smooth surface morphology that reduces the leakage current, and (iii) the X-F6-TAPC layer effectively reduces the hole injection barrier between PEDOT:PSS and EML.

An important consequence of the above three factors is the reduction of the Joule heating effect that reduces the LED operational temperature. To this end, we have compared surface temperatures of our control (w/o HTL) and X-F6-TAPC based devices using an infrared camera. Upon operation at a relatively high voltage, 5.5 V, the LED surface temperature increased from 22.7 to 30.7 and 27.8 °C, respectively, as shown in Supplementary Figure 42. We conclude that the reduced Joule heating effect is responsible for the improved operational lifetime of LEDs.

Supplementary Tables

Supplementary Table 1. Photophysical characteristics of the LHP ANCs thin films deposited on bare glass substrate and HTL, poly-TPD and X-F6-TAPC.

LHP ANCs film on	η_{PL} (%)	$\lambda_{max.}$ (nm)	fwhm (nm)	τ_{avg} (ns)	t_1 (ns)	t_2 (ns)
bare glass	98.2 ± 1.8	528.9	22.8	51.1	19.5	78.2
poly-TPD layer	45.7 ± 1.8	527.3	23.3	44.2	18.5	68.9
X-F6-TAPC layer	95.3 ± 1.3	527.4	23.2	50.0	19.2	75.8

Supplementary Table 2. Sellmeier parameters obtained from fitting the SE data for LHP ANCs, cross-linkable hole transporting material (HTM), X-F6-TAPC, and a conventional HTM, Poly-TPD.

Material	A_1 (-)	A_2 (-)	A_3 (-)	B_1 (μm^2)	B_2 (μm^2)	B_3 (μm^2)
LHP ANCs	1.49513	1.181517	5.38914	0.05010	30180.03125	11148.9932
X-F6-TAPC	1.29824	6412.6567	1013.648	0.05116	39260.57031	2950.19287
Poly-TPD	0.94802	0.0186308	1.610195	0.11773	30184.38281	11170.6973

Supplementary Table 3. EL characteristics of perovskite quantum dot LEDs.

Device	V_{on} (V)	η_{CE} (cd A^{-1})	η_{ext} (%)	λ_{max} (nm)
ITO/PEDOT:PSS/$FA_{0.5}MA_{0.5}PbBr_3$/TPBi/LiF/Al	2.75	24.74	5.87	528
ITO/PEDOT:PSS/$FA_{0.5}MA_{0.5}PbBr_3$/3TPYMB/LiF/Al	2.75	37.86	9.44	528
ITO/PEDOT:PSS/$FA_{0.5}MA_{0.5}PbBr_3$/3TPYMB/Liq/Al	2.75	46.60	10.6	528
ITO/PEDOT:PSS/ X-F6-TAPC/$FA_{0.5}MA_{0.5}PbBr_3$/PO-T2T/Liq/Al	3.00	42.52	10.34	528
ITO/PEDOT:PSS/X-F6-TAPC/$FA_{0.5}MA_{0.5}PbBr_3$/B3PYMB/Liq/Al	3.00	36.11	8.69	528
ITO/PEDOT:PSS/X-F6-TAPC/$FA_{0.5}MA_{0.5}PbBr_3$/3TPYMB/Liq/Al	2.80	103.4	24.96	528
ITO/PEDOT:PSS/Poly-TPD/X-F6-TAPC/$FA_{0.5}MA_{0.5}PbBr_3$/ 3TPYMB/Liq/Al	3.00	35.6	8.6	528

Supplementary Table 4. Summary of perovskite LEDs made by EMLs with preferentially horizontal-oriented TDMs, comparing with our results.

Emitter	η_{PL} (%)	λ_{EL} (nm)	Θ_H (%)	η_{ext} (%)	Ref.
$PBABr_y(Cs_{0.7}FA_{0.3}PbBr_3)$	60	483	NA*	9.5	[49]
ANCs	98	528	0.73±0.016	24.96	This work

*Quantitative Θ_H values was not reported.

Supplementary Figures

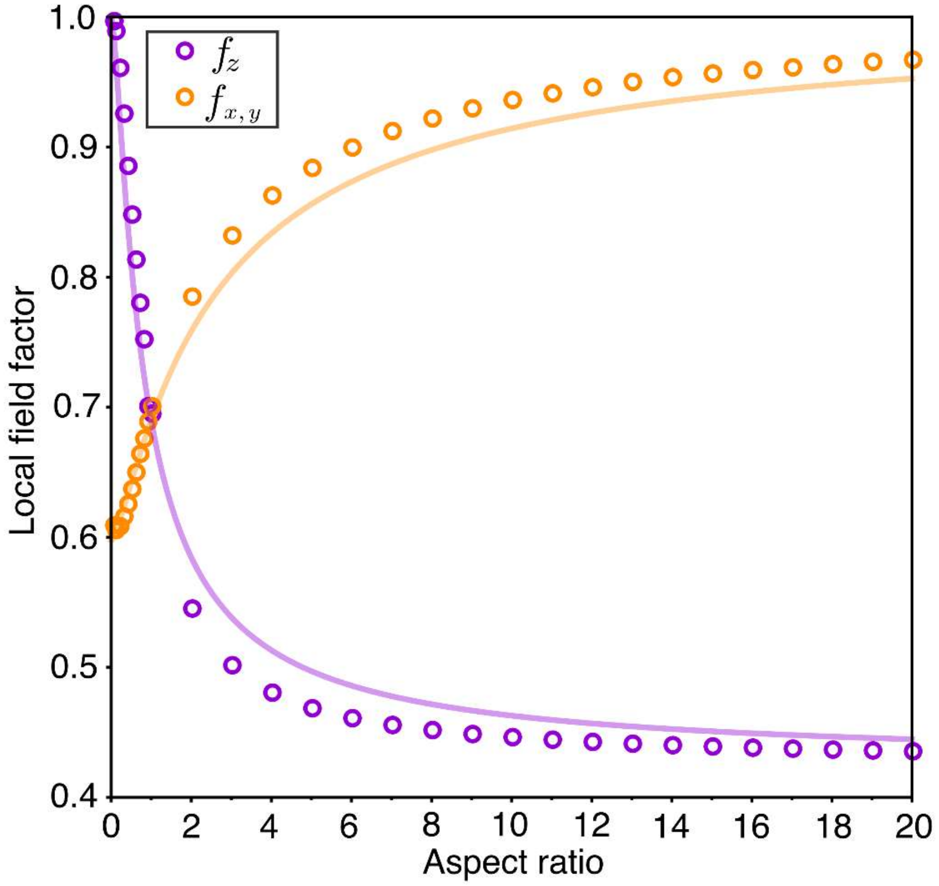

Supplementary Figure 1. Local field factors as a function of the aspect ratio. The solid lines correspond to the analytical solutions for the spheroids eq. (5), and the open circles are the numerical solutions for cuboids.

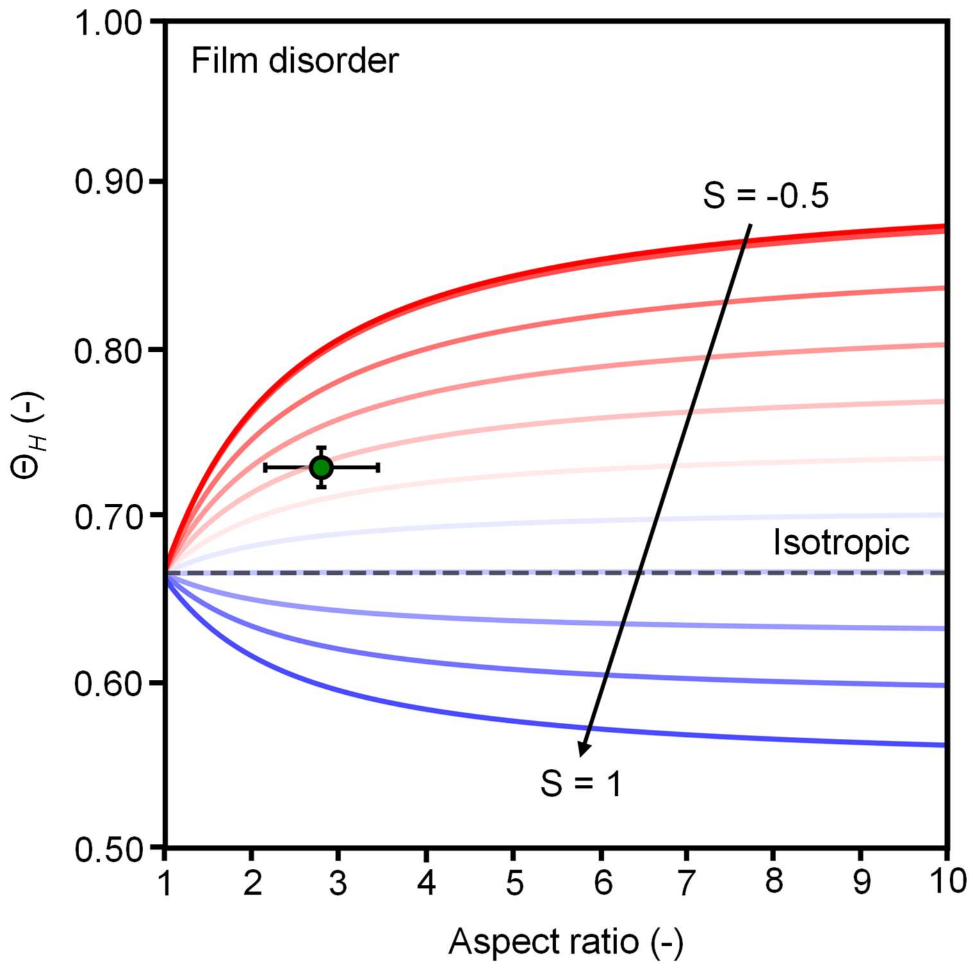

Supplementary Figure 2. Effect of disorder in film on directed emission of ANCs. The top red line corresponds to the single NC limit and all the other curves are the result of orientational averaging according to different orientational distributions. The green mark represents the average experimental measurement.

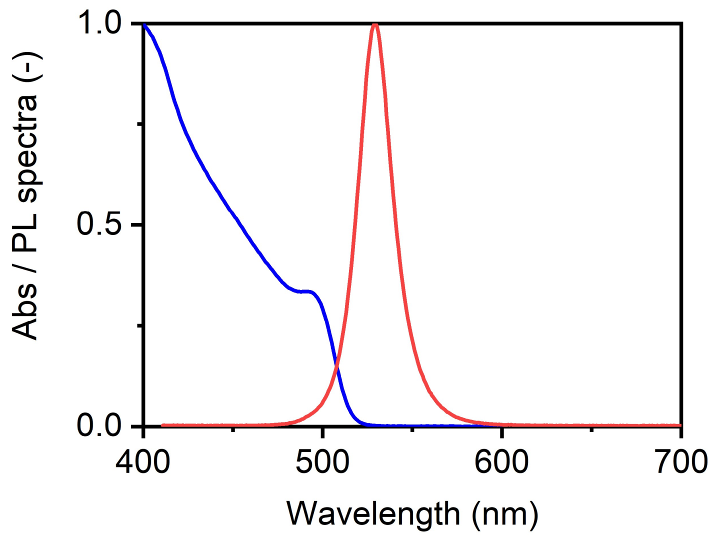

Supplementary Figure 3. The PL (red line) and absorption (Abs.; blue line) spectra of colloidal LHP NCs recorded in toluene.

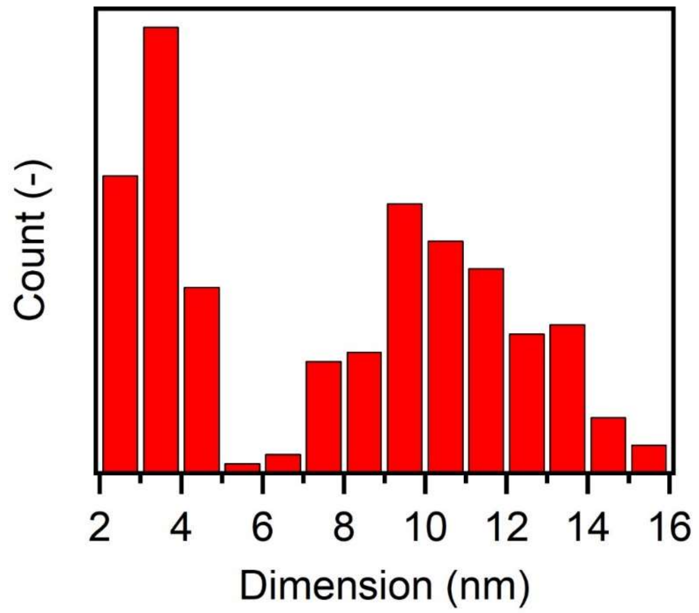

Supplementary Figure 4. Histograms of lateral size distribution by analyzing the cryo-TEM micrograph.

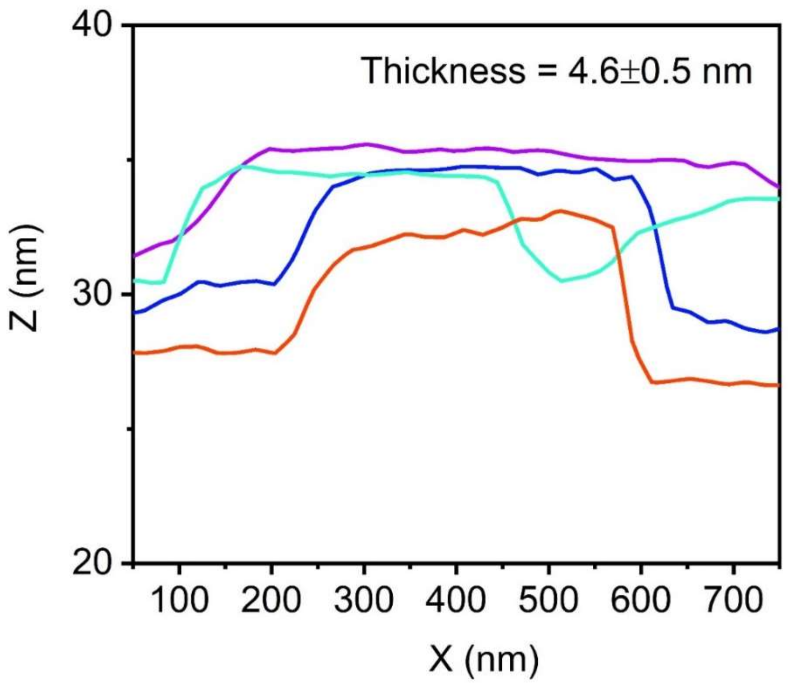

Supplementary Figure 5. Cross-sectional height profiles of ANSLs in the *xy* (substrate) plane, in AFM height image presented in Figure 2b.

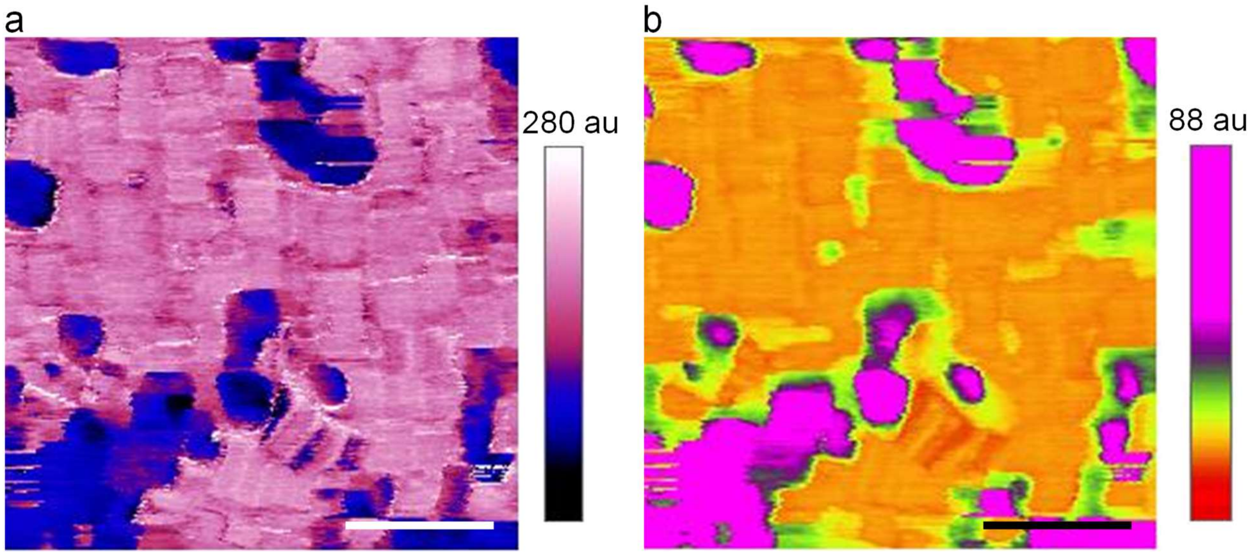

Supplementary Figure 6. Different AFM mode images of our anisotropic LHP NC film **a.** Derjaguin-Muller-Toporov (DMT) Young's modulus mode considering the load force and adhesion. **b.** Adhesion mode. (scale bar = 0.5 μm).

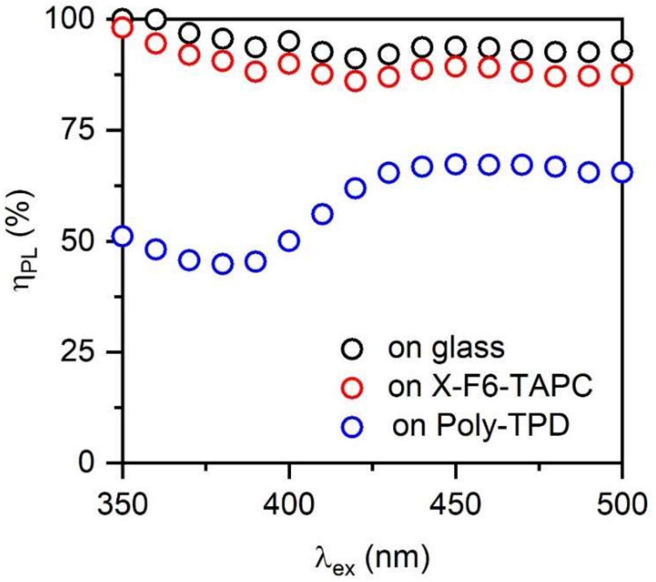

Supplementary Figure 7. The η_{PL} of anisotropically confined LHP ANCs on the glass, X-F6-TAPC, and Poly-TPD thin films by varying excitation wavelengths (λ_{ext}) from 350 to 500 nm.

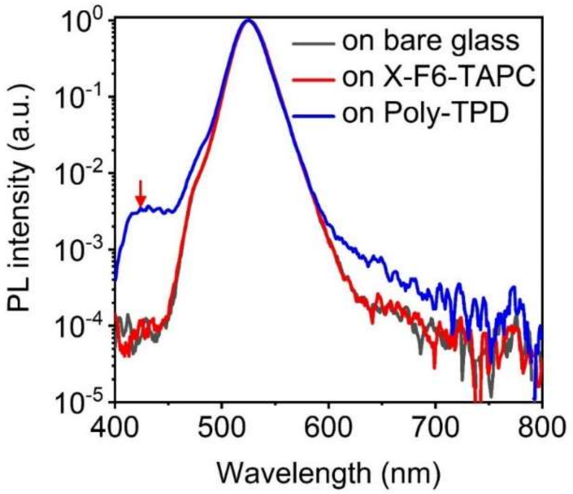

Supplementary Figure 8. PL spectra of anisotropically confined LHP ANCs solid thin films on the bare glass, X-F6-TAPC, and Poly-TPD thin films.

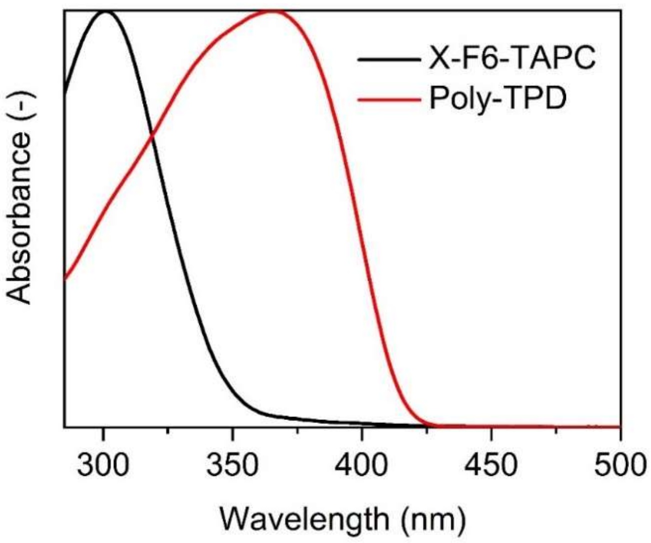

Supplementary Figure 9. Absorbance spectra of X-F6-TAPC (black line) and Poly-TPD (red line) hole transporting layers.

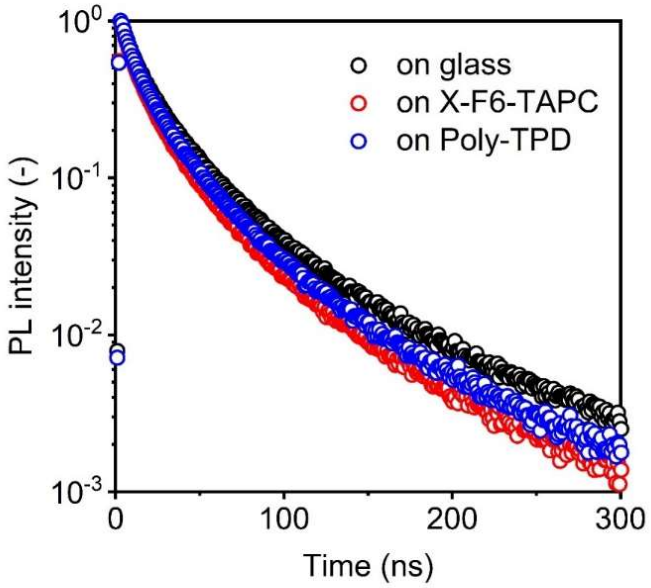

Supplementary Figure 10. Time resolved PL spectra of anisotropically confined LHP ANCs thin films on the bare glass substrate (black circle), X-F6-TAPC (red circle), and Poly-TPD (blue circle) thin films.

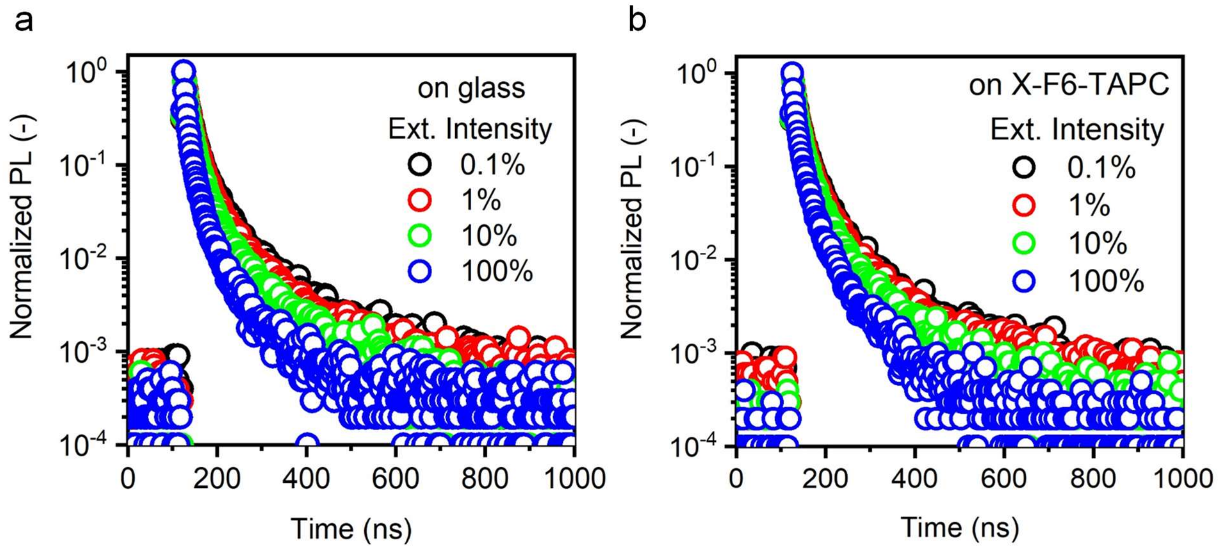

Supplementary Figure 11. Excitation power-dependent TRPL spectra of ANC thin films on **a.** glass substrate and **b.** X-F6-TAPC layer.

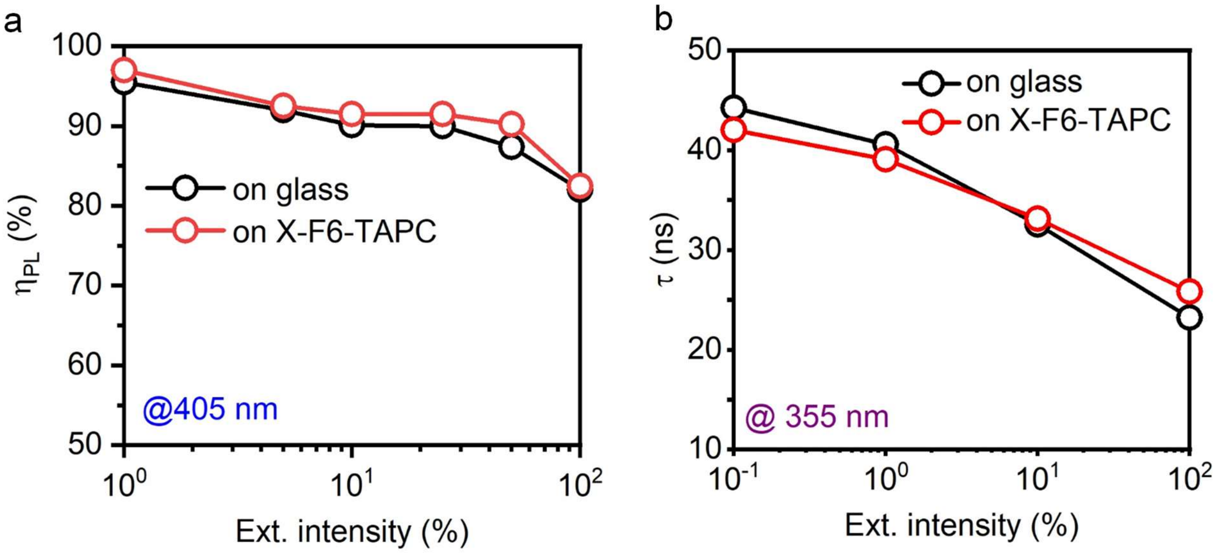

Supplementary Figure 12. a. η_{PL} as a function of excitation power. **b.** PL lifetime as a function of excitation power.

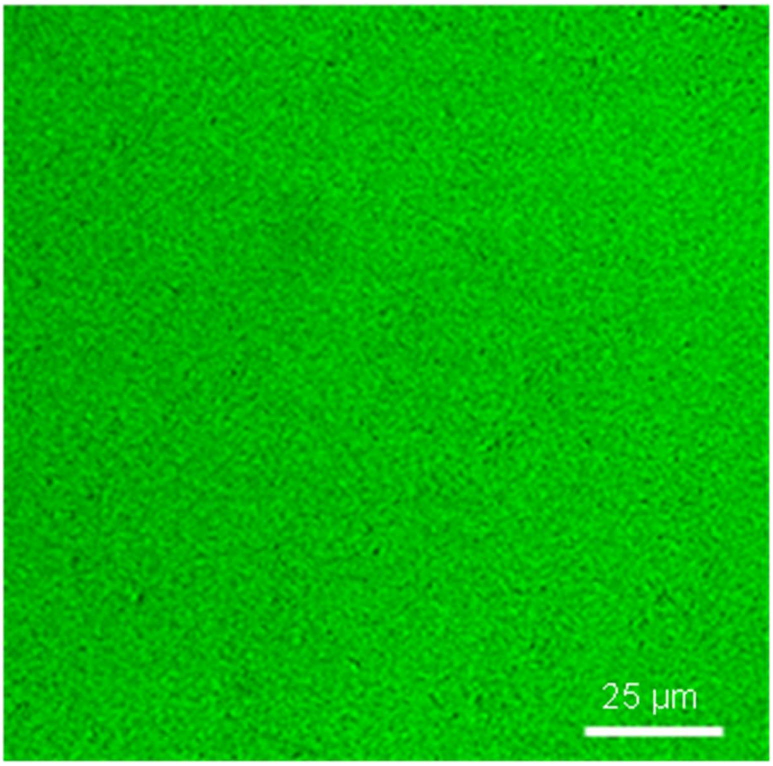

Supplementary Figure 13. Scanning PL micrograph of LHP nanocrystal thin film deposited on X-F6-TAPC HTL taken by a confocal Olympus FluoView 3000 microscope.

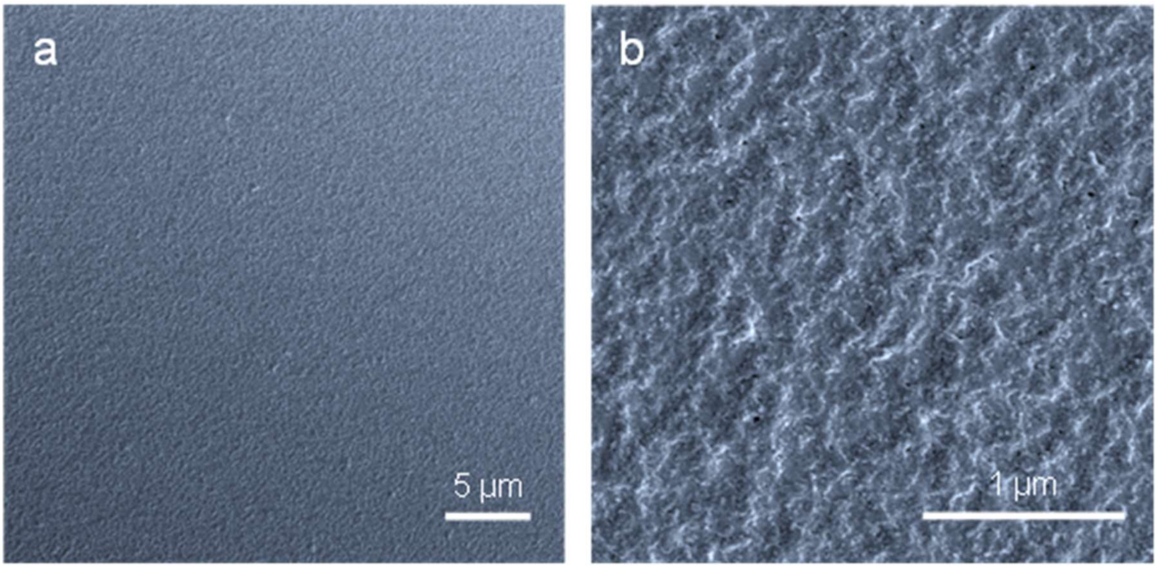

Supplementary Figure 14. SEM images showing surface morphology of our ANC EML at a horizontal field width of **a.** 59.2 µm and **b.** 5.0 µm.

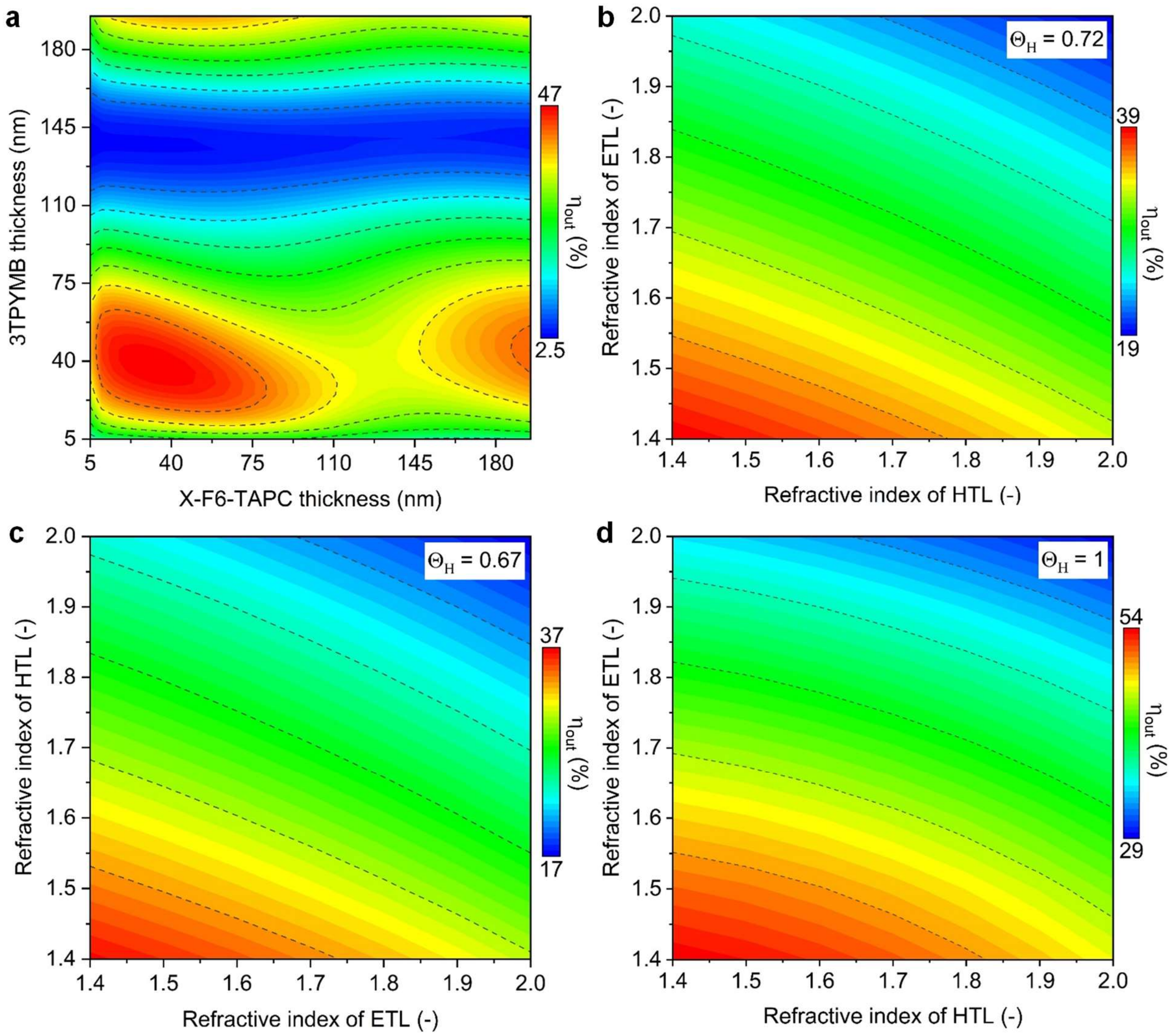

Supplementary Figure 15 a. Contour plot of maximum achievable light extraction efficiency (η_{out}) distribution as a function of electron transporting layer, 3TPYMB, and hole transporting layer, X-F6-TAPC, thicknesses. Contour plot of maximum achievable light extraction efficiency (η_{out}) distribution as a function of TDM orientation, **b.** Θ_H = 0.72 (experimental), **c.** Θ_H = 0.67 (isotropic), and **d.** Θ_H = 1 by varying the refractive indices of HTLs and ETLs between 1.4 and 2.0.

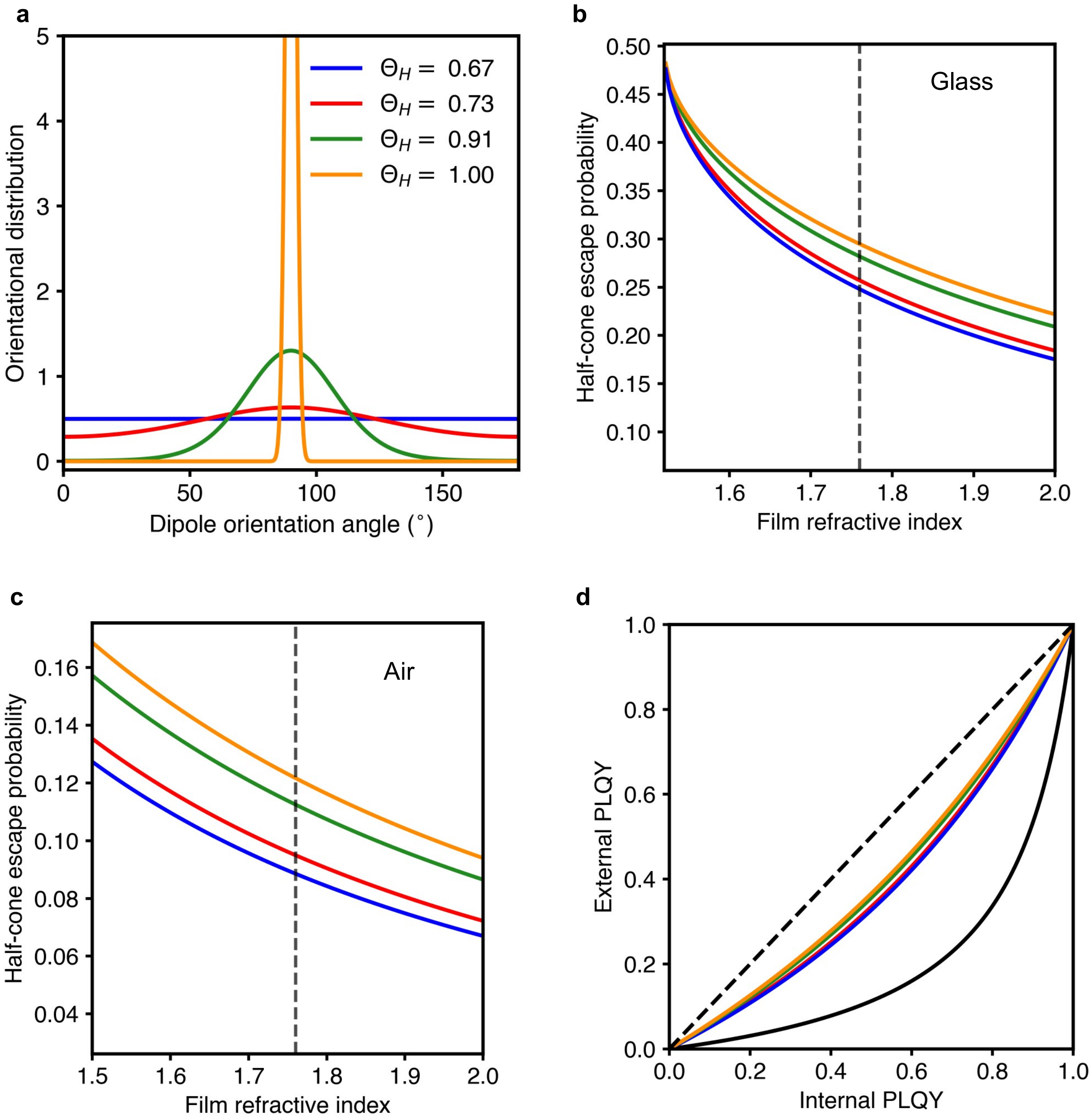

Supplementary Figure 16. The effects of photon recycling and TDM orientation on the external photoluminescence quantum yields. **a.** Maximum entropy dipole orientational distribution corresponding to different Θ_H values. **b-c.** Half-cone escape probabilities as a function of the refractive index of the emissive film towards air (**b**) and glass (**c**) respectively. The different solid lines correspond to the same dipole orientation values in panel **a.** The dashed line represents the refractive index of EML in our LEDs in the main text. **d.** Calculated internal η_{PL} versus external η_{PL} values for different total escape probabilities taking into account photon recycling. The black solid line corresponds a value of escape probability of 12.7% as used in Ref. 26.

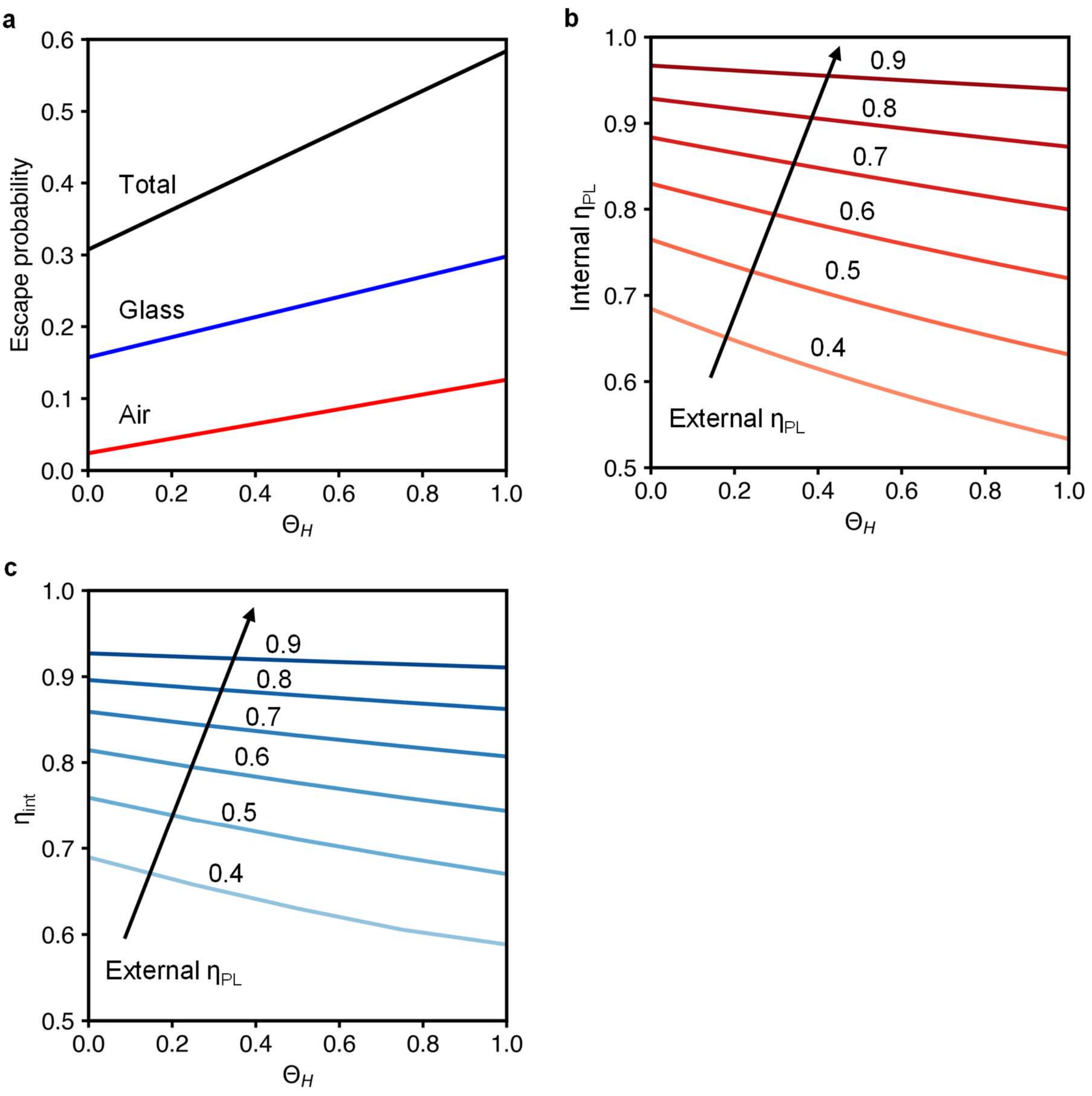

Supplementary Figure 17. Calculation of device internal quantum efficiency η_{int} in actual LED device architecture. **a.** Calculated escape probabilities as a function of Θ_H. **b-c.** Internal η_{PL} and η_{int} as a function of dipole orientation Θ_H for different external η_{PL} values.

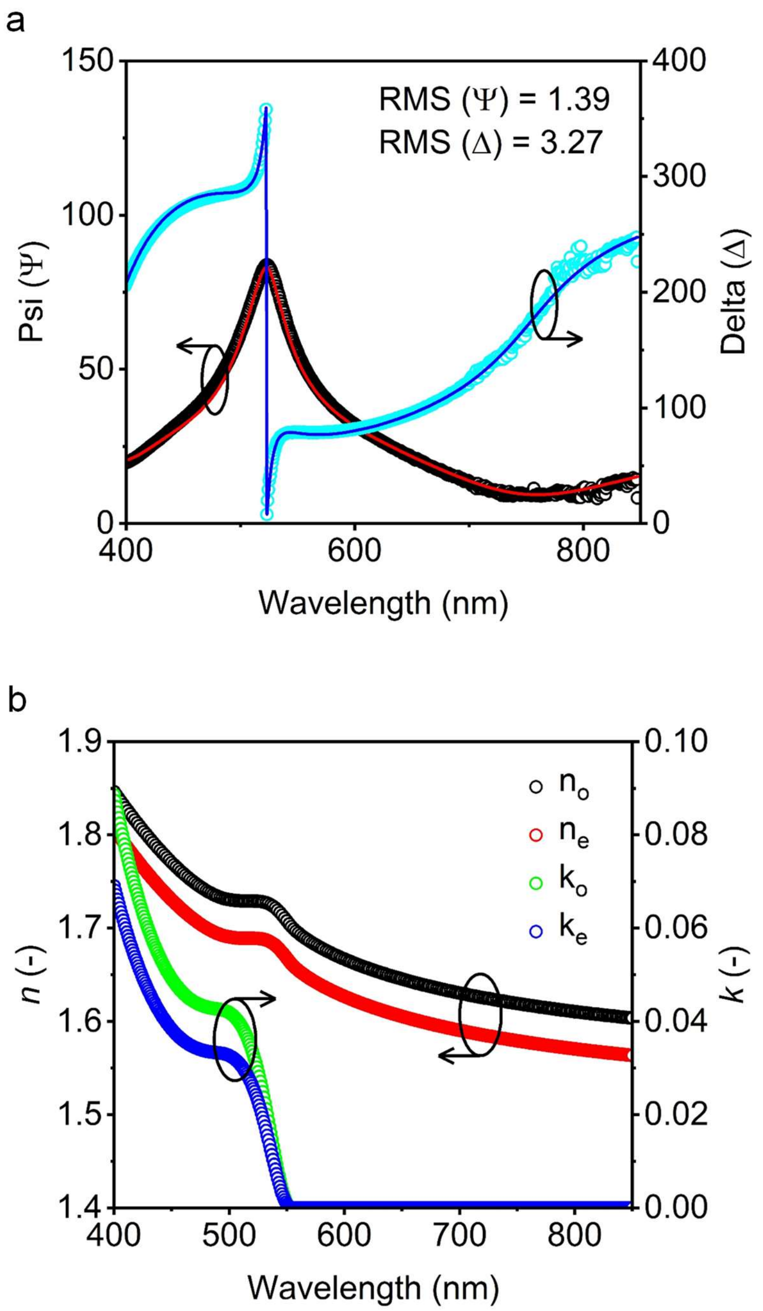

Supplementary Figure 18 a. Ψ and Δ experimental data and corresponding Tauc-Lorentz fittings for our LHP nanocrystal EML assuming isotropic refractive index and zero surface roughness. The excellent fittings suggest that both roughness and birefringence are both negligible. **b.** The calculated Tauc-Lorentz dispersions for n_e, n_o, k_e, and k_o assuming the maximum degree of birefringence for S = -0.08. Using the dispersions in optical simulations, the calculated outcoupling efficiency only changes by 0.5%, as compared to isotropic model.

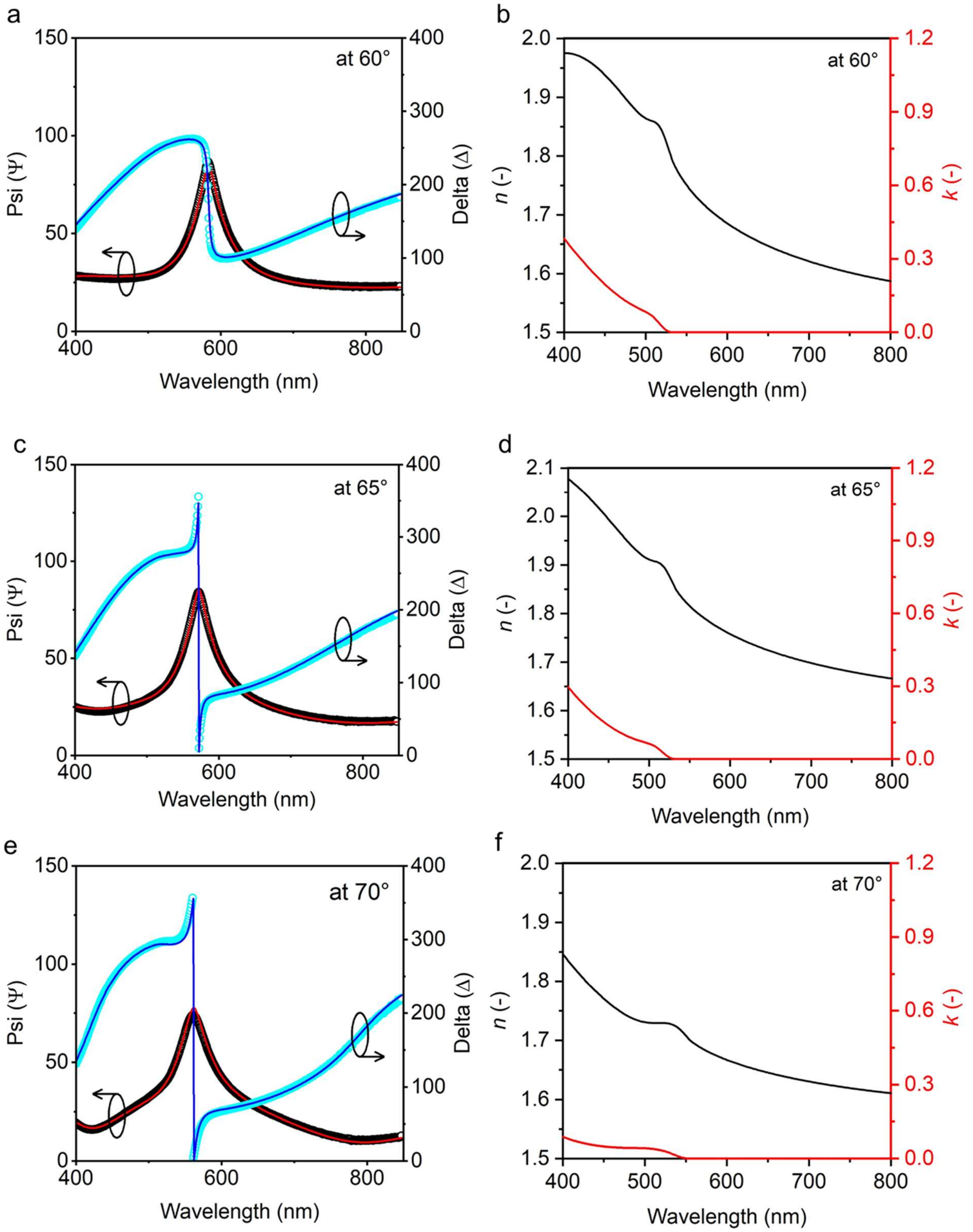

Supplementary Figure 19. Variable angle spectroscopic ellipsometry (VASE) analysis. Spectroscopic ellipsometry Ψ and Δ experimental data and corresponding Tauc-Lorentz fitting for ANSL films *n* and *k* values at 60º (**a** and **b**), 65º (**c** and **d**), and 70º (**e** and **f**).

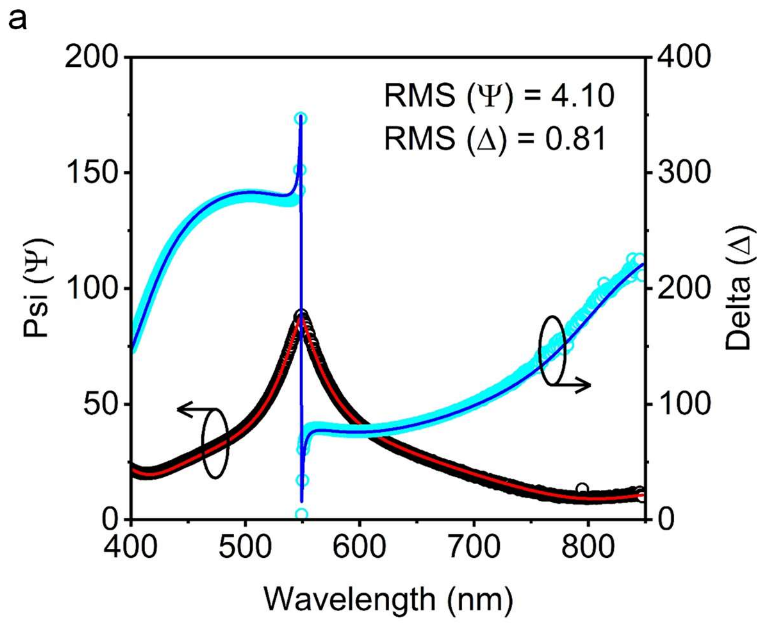

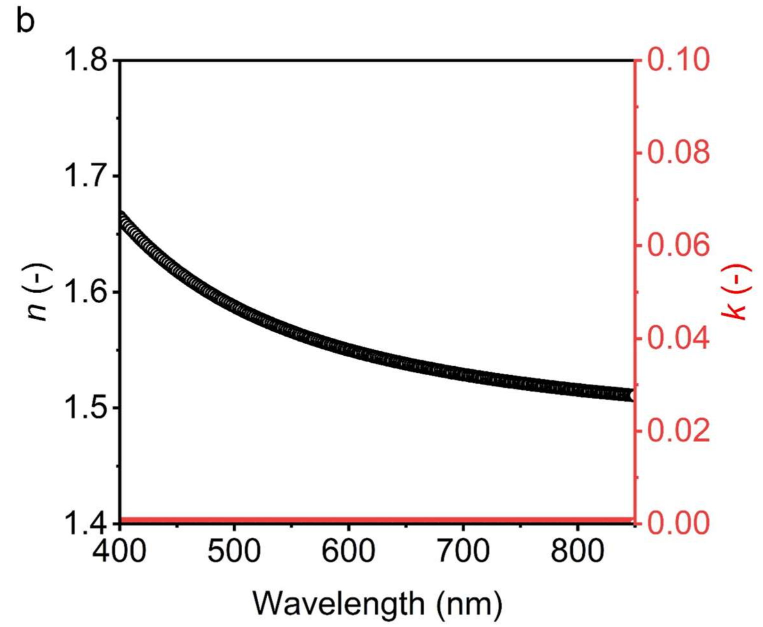

Supplementary Figure 20 a. Ψ and Δ experimental data and corresponding Tauc-Lorentz fitting for our X-F6-TAPC HTL. **b.** The calculated Tauc-Lorentz dispersion relations for its n and k used in optical simulations.

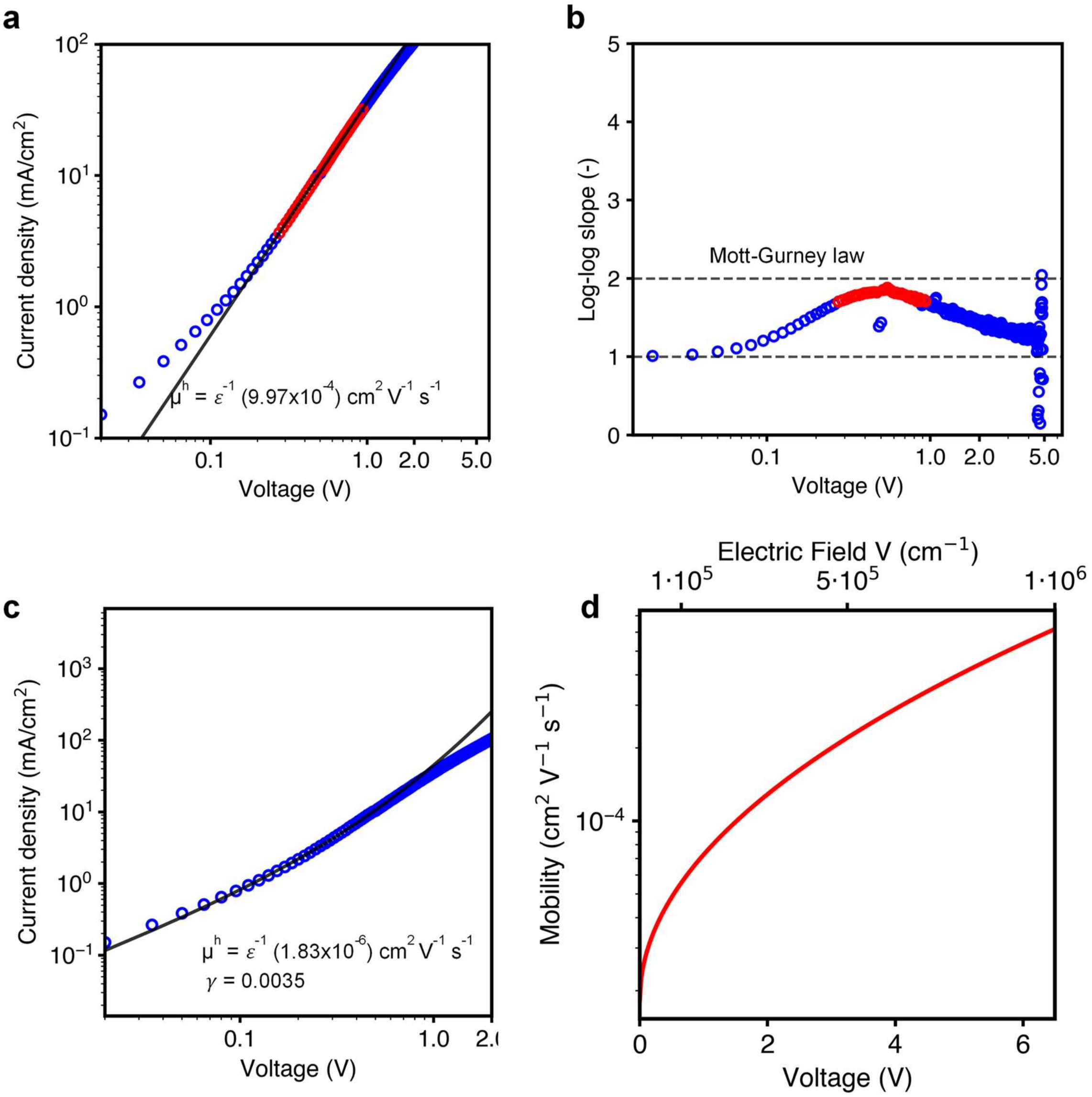

Supplementary Figure 21 a. *J-V* characteristics of the hole-only device. The red points correspond to the defined SCLC regime and the black curve is the corresponding Mott-Gurney fit. **b.** Slop of the log(J)-log(V) plot used to determine the SCLC regime. **c.** *J-V* curve with the improved field-dependent mobility fit. **d.** Plot of the field dependence of hole mobility.

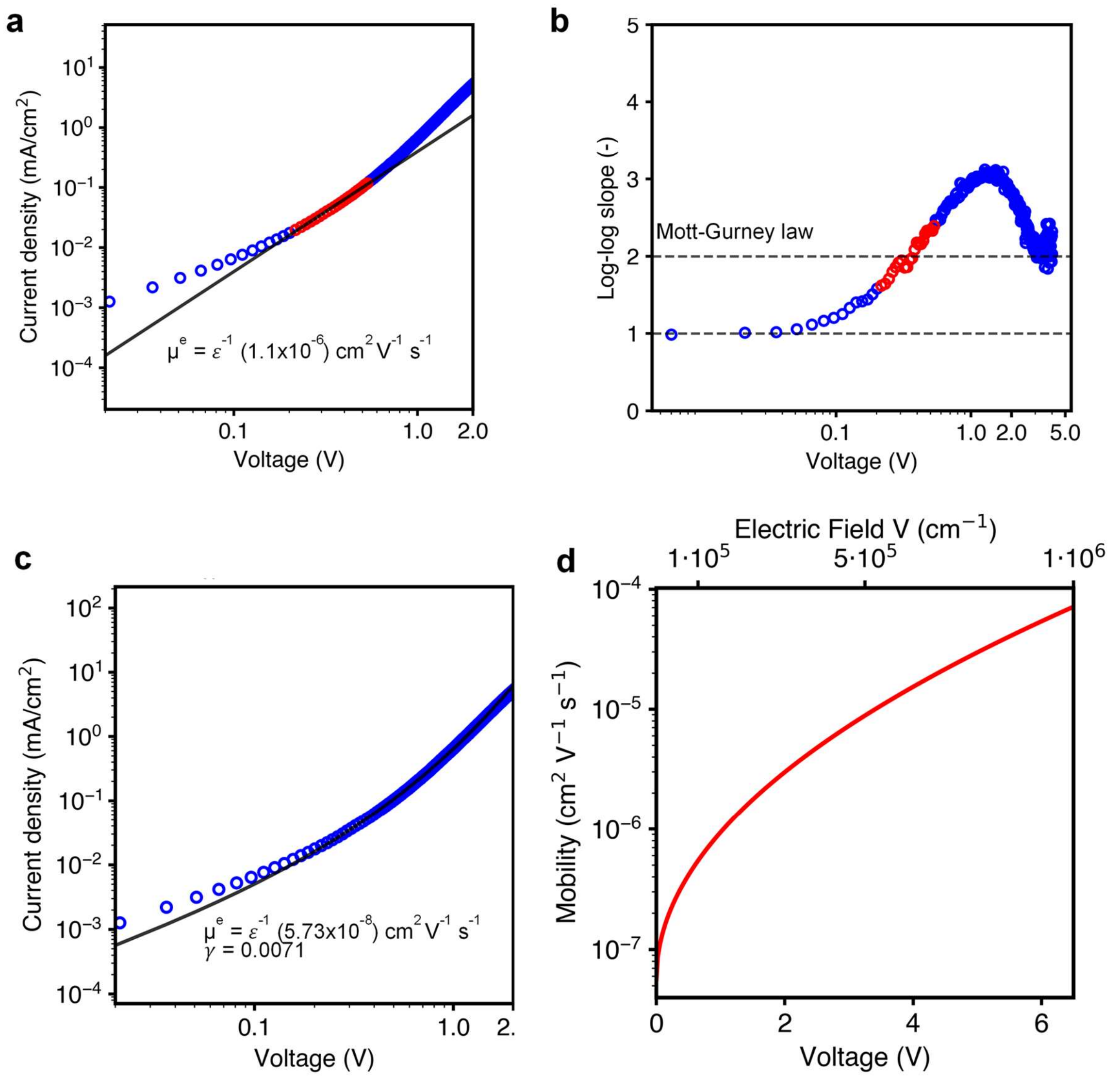

Supplementary Figure 22 a. *J-V* characteristics of the electron-transport-only device. The red points correspond to the defined SCLC regime and the black curve is the corresponding Mott-Gurney fit. **b.** Slop of the log(J)-log(V) plot used to determine the SCLC regime. **c.** *J-V* curve with the improved field-dependent mobility fit. **d.** Plot of the field dependence of electron mobility.

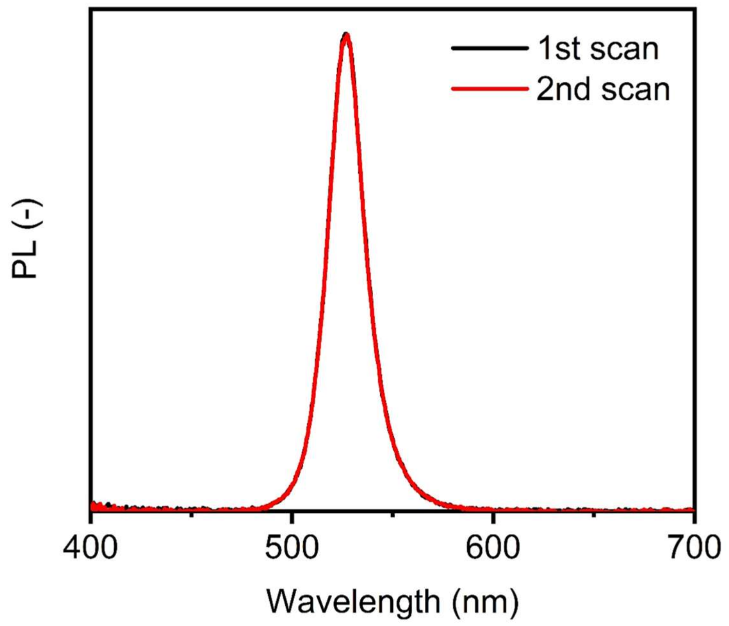

Supplementary Figure 23. PL spectra of our perovskite NC thin film at $\phi = 0°$ from two separate sweepings of polarization angles recorded at the same spot.

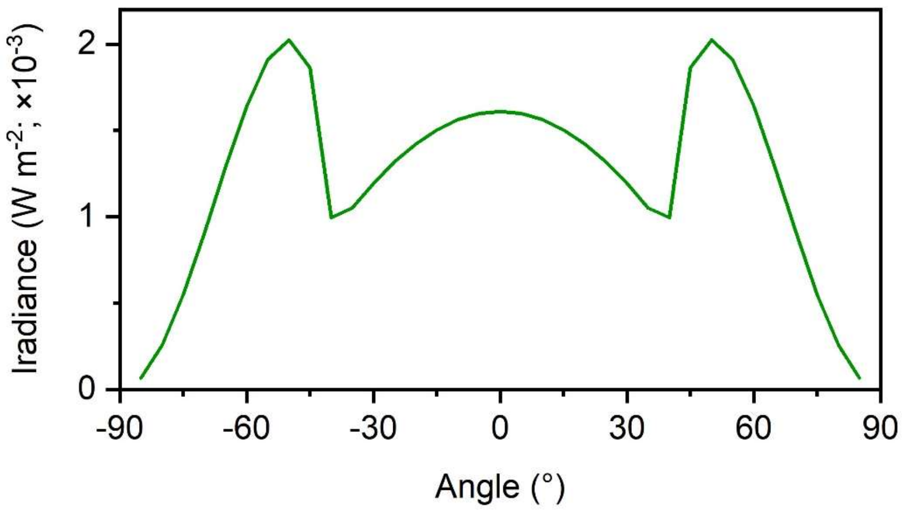

Supplementary Figure 24. Representative full-range angular measurement [-85°, 85°] recording the PL irradiance of our NC thin-film sample.

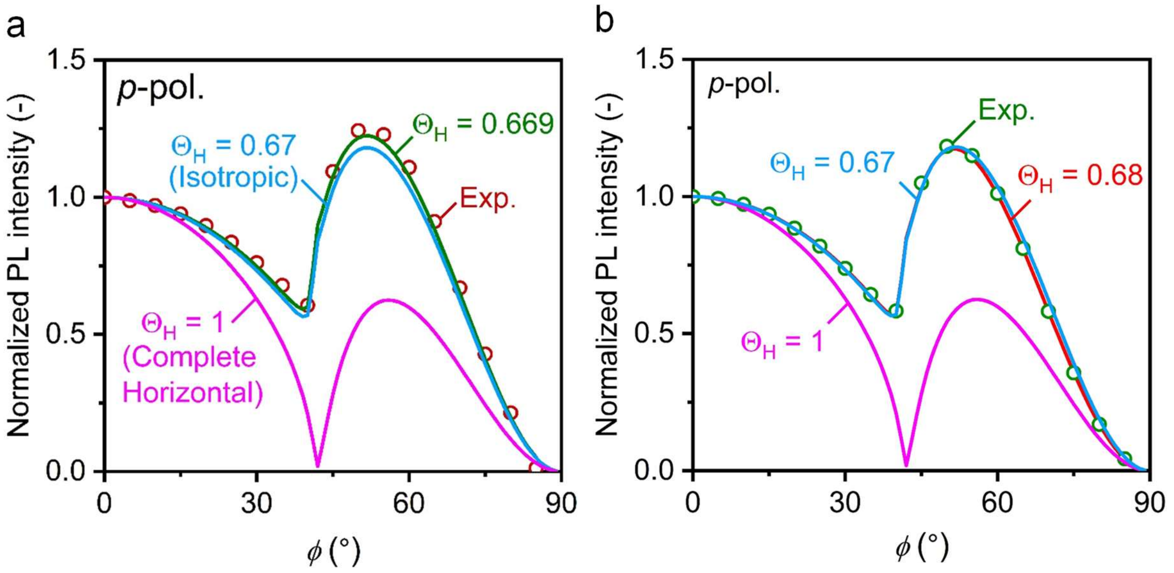

Supplementary Figure 25. Experimentally characterized (open circles; Exp.) and optical theory calculated thin-film *p*-pol radiation patterns for our perovskite anisotropic NCs deposited on, **a.** bare glass, and **b.** Poly-TPD layer.

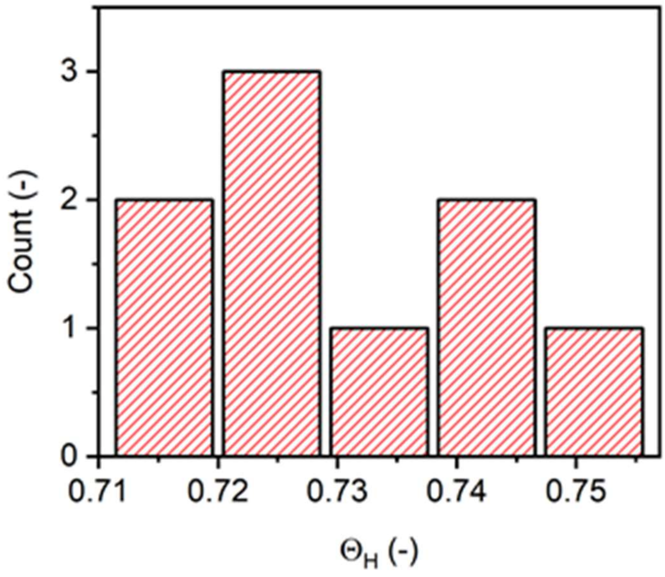

Supplementary Figure 26. Statistical distribution of Θ_H of various LHP ANC thin films having a layer sequence of LHP film/X-F6-TAPC/glass.

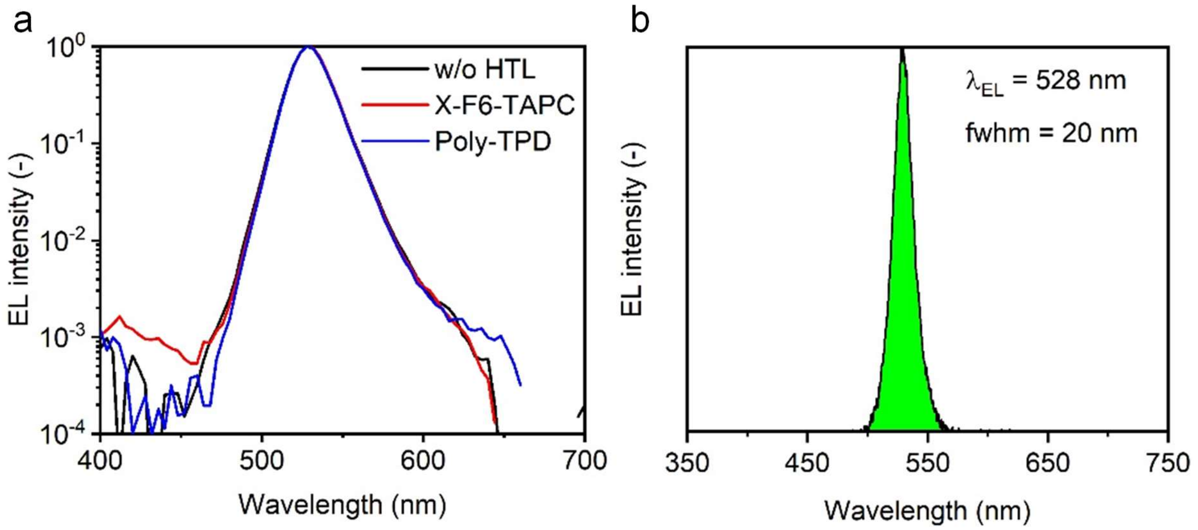

Supplementary Figure 27a. EL spectra of devices, either without (w/o) HTL or having X-F6-TAPC, and poly-TPD as HTL. **b.** EL spectra of optimal perovskite QD LEDs demonstrating ultra-pure green emission with $CIE_{x,y}$ coordinates of (0.174, 0.795).

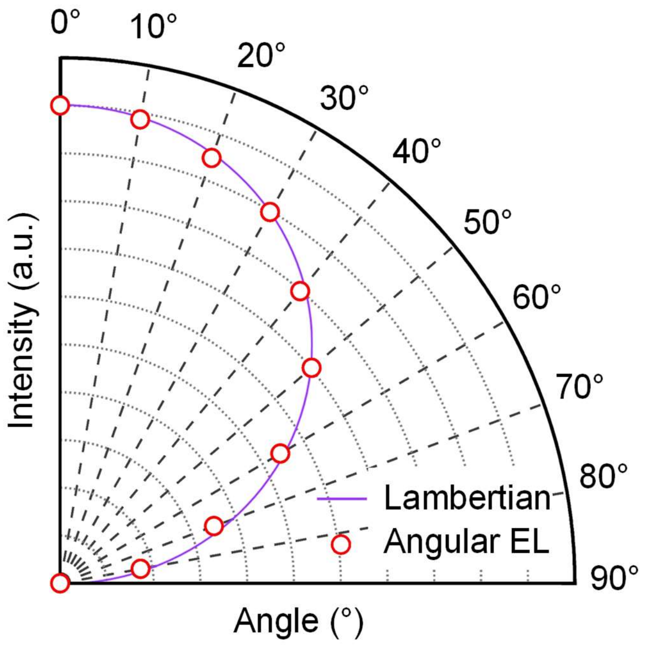

Supplementary Figure 28. Distribution of EL emission intensities of the perovskite QD LED device by varying the viewing angles between 0° and 90°.

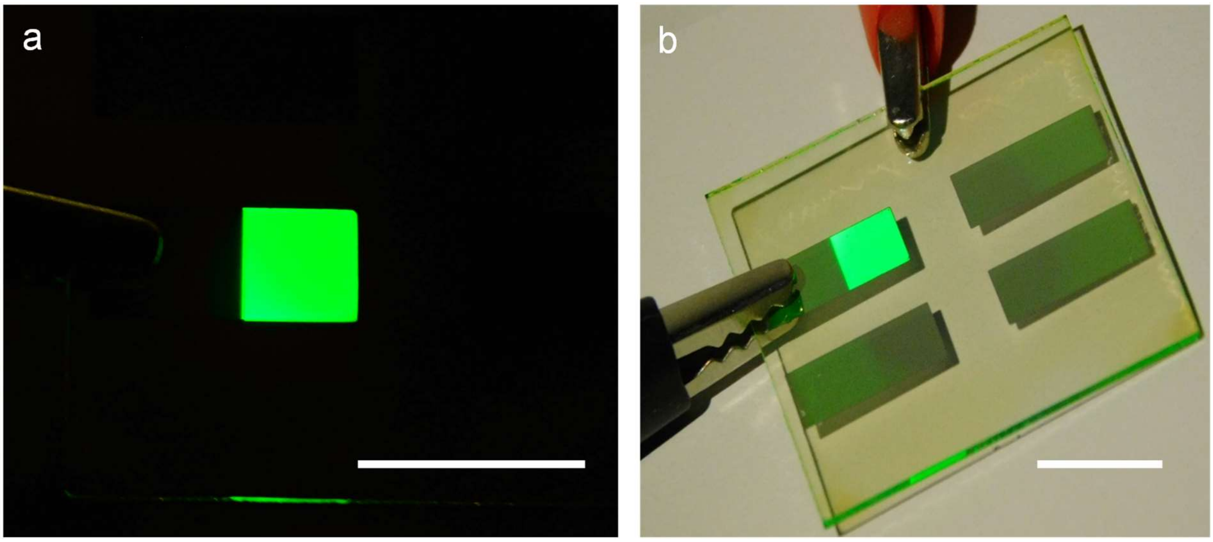

Supplementary Figure 29. Photographs of a LED device emitting, **a.** at ~8 cd m^{-2} (in dark) and **b.** at 55 cd m^{-2} (in ambient light). Scale bars: 1 cm.

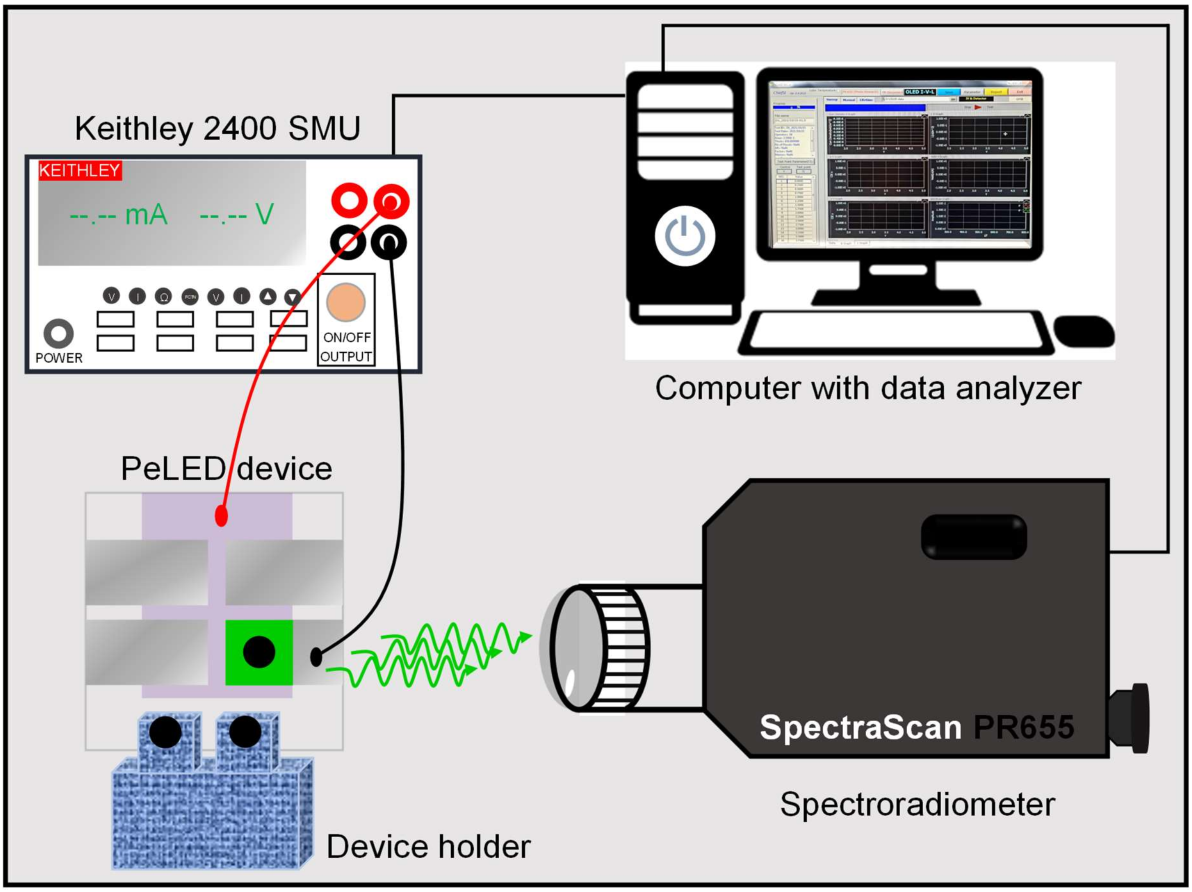

Supplementary Figure 30. Schematic diagram of the EQE measurement set-up.

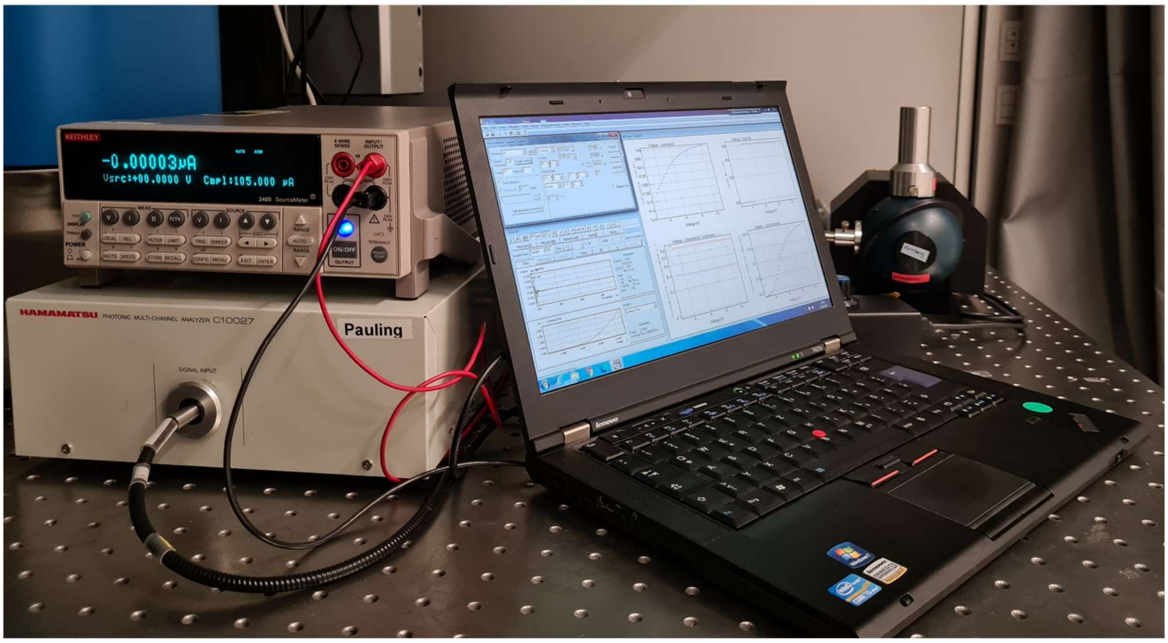

Supplementary Figure 31. Photograph of absolute η_{ext} measurement system (C9920-12) from Hamamatsu Photonics.

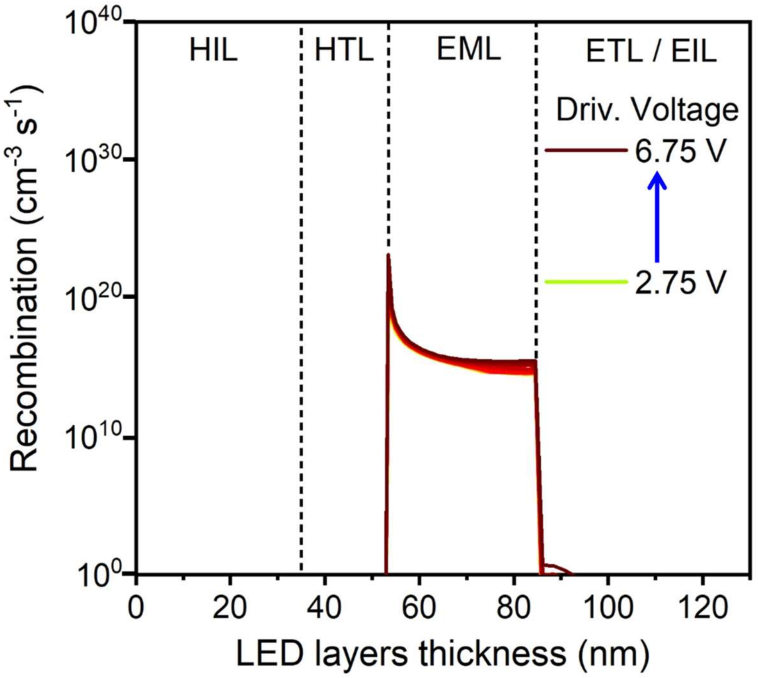

Supplementary Figure 32. Recombination profile across the layers of the X-F6-TAPC based perovskite LED device.

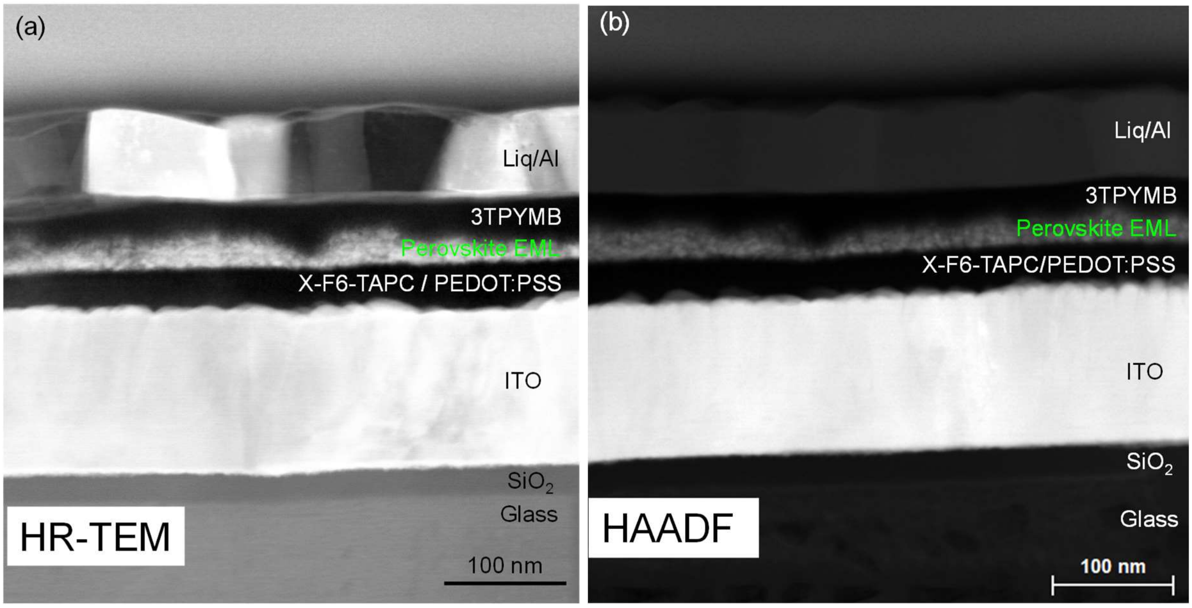

Supplementary Figure 33. Cross-sectional (a) HR-TEM and (b) HAADF-STEM images for our optimized device.

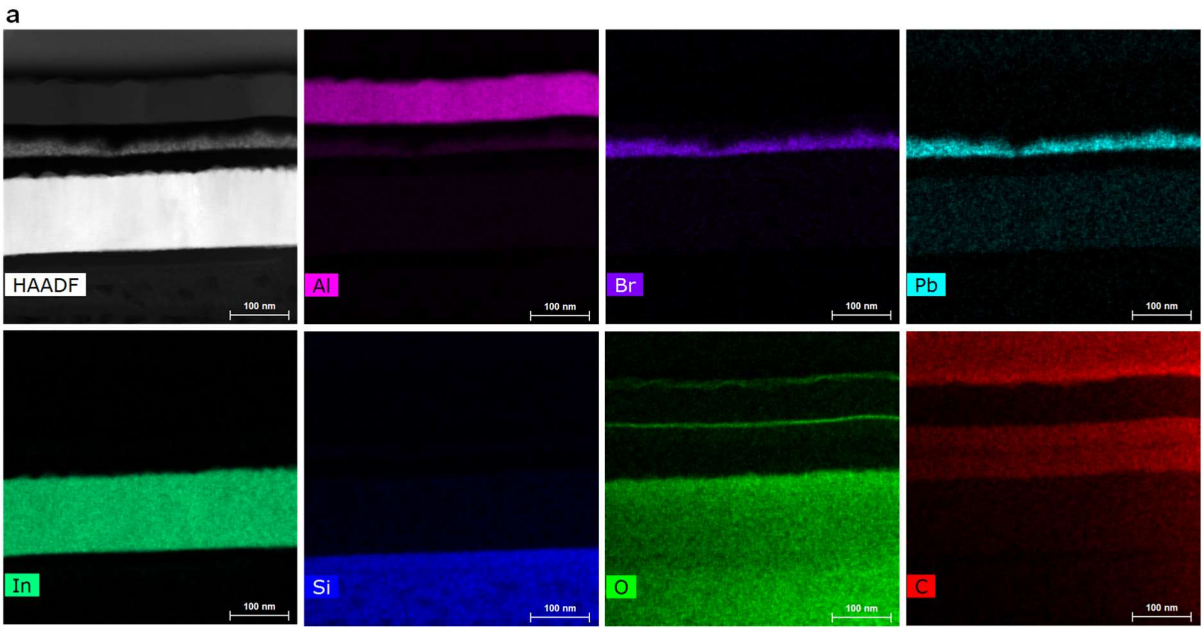

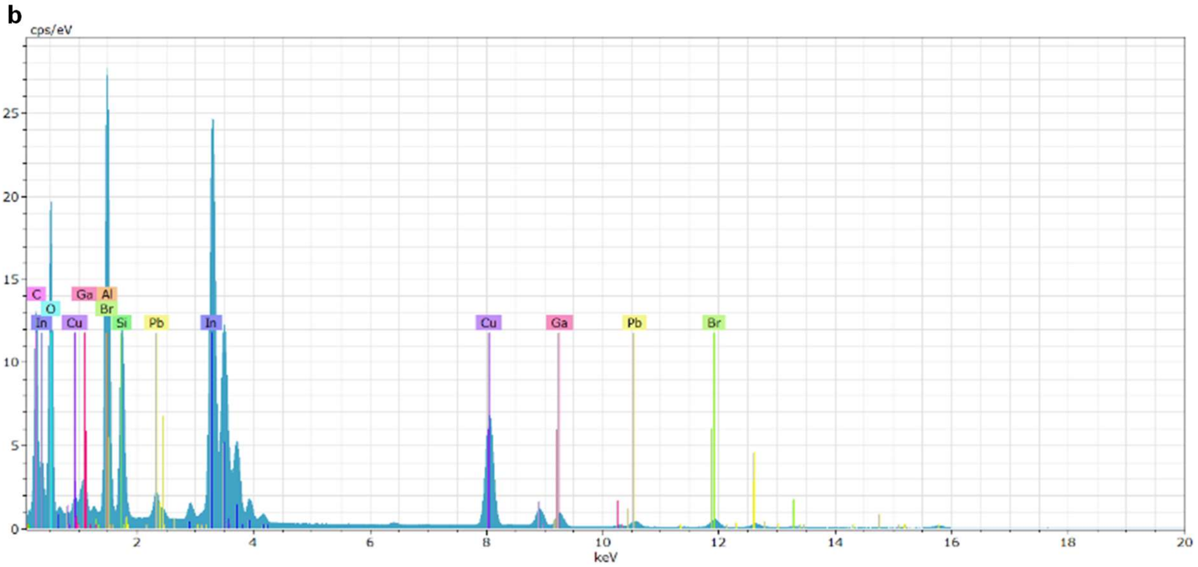

Supplementary Figure 34 a. Highlight of HAADF scanning TEM image of optimal device cross-section with area highlighted for EDX mapping. **b.** EDX spectra of device cross-section showing all elements.

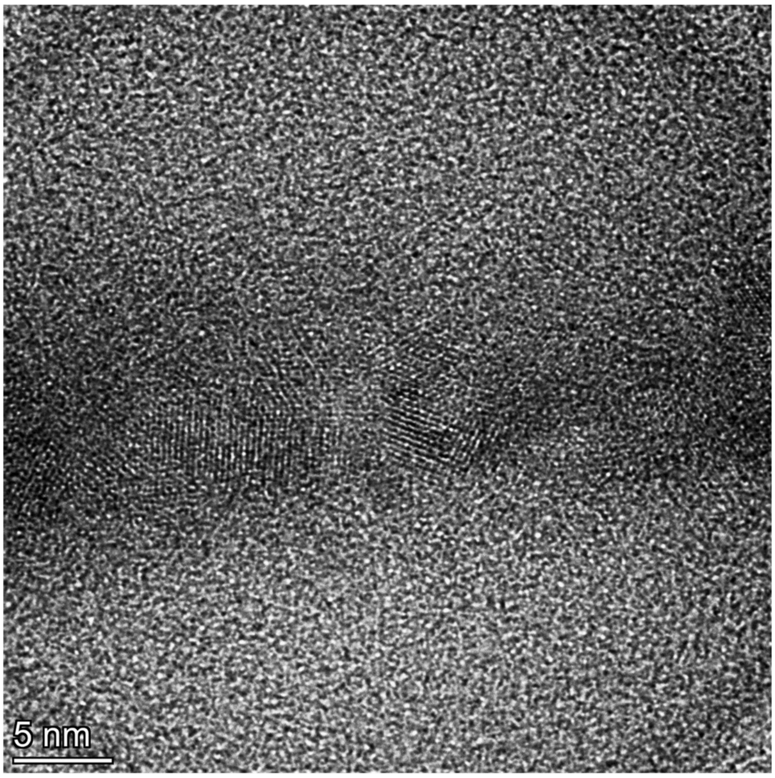

Supplementary Figure 35. HR-TEM image of LHP anisotropic NCs.

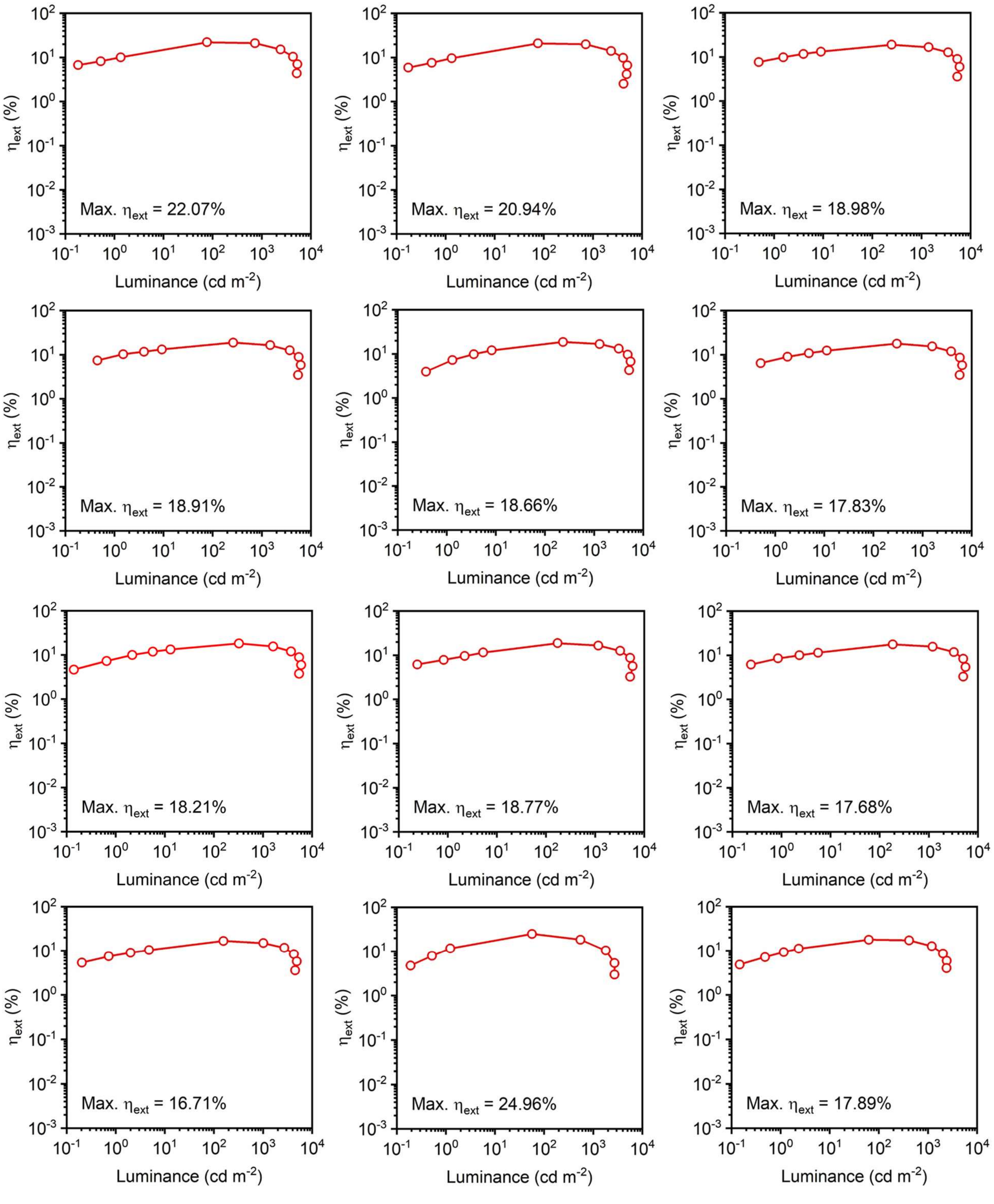

Supplementary Figure 36. The characterized external quantum efficiency as a function of luminance in our efficiency champion device, ranging the highest EQE between 16.71% and 24.96%.

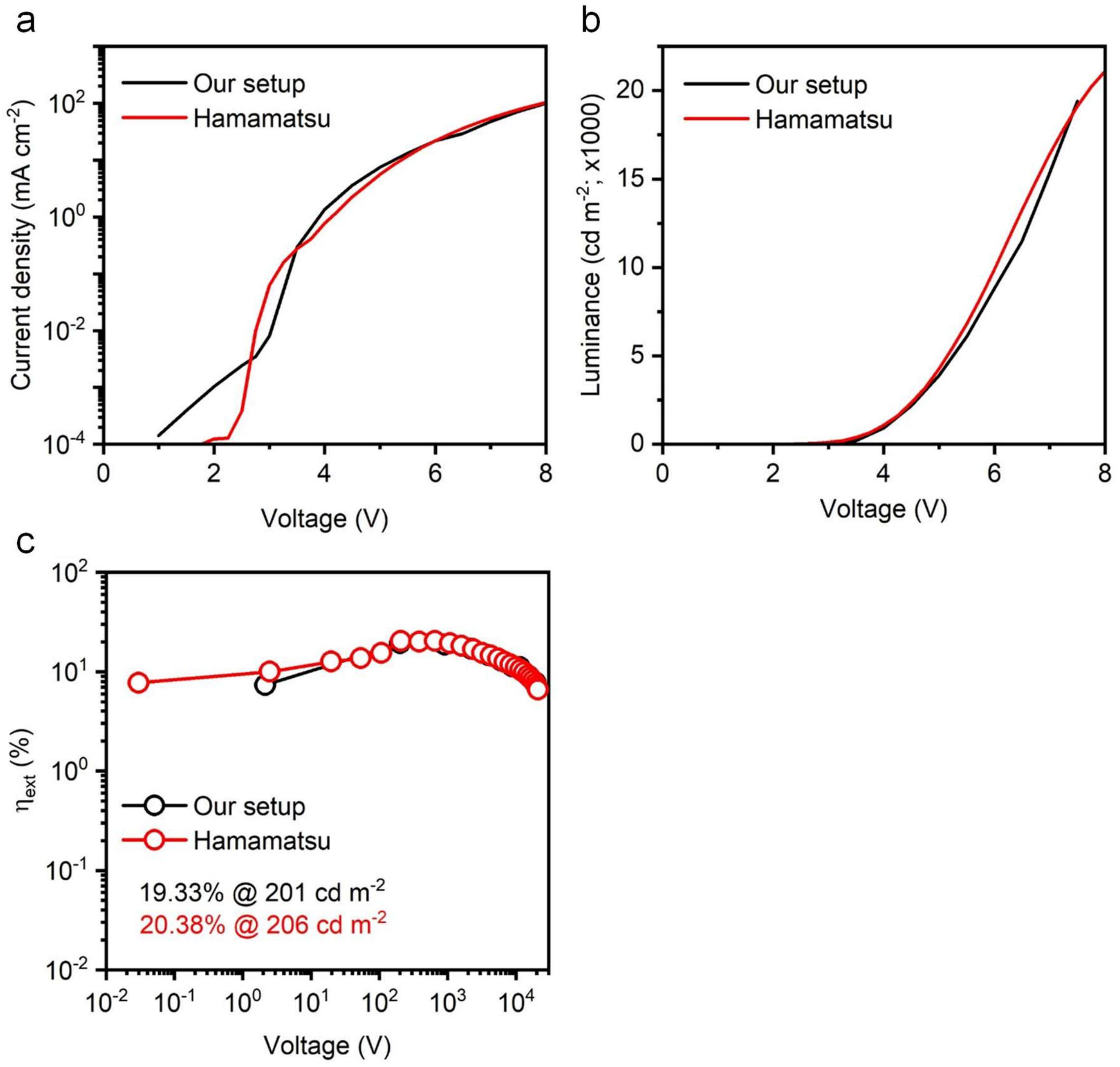

Supplementary Figure 37. Comparison of characterized η_{ext} of as a function of luminance for representative OLED devices using our setup and a calibrated commercial EQE measurement system located in Hamamatsu Co. Ltd. Germany.

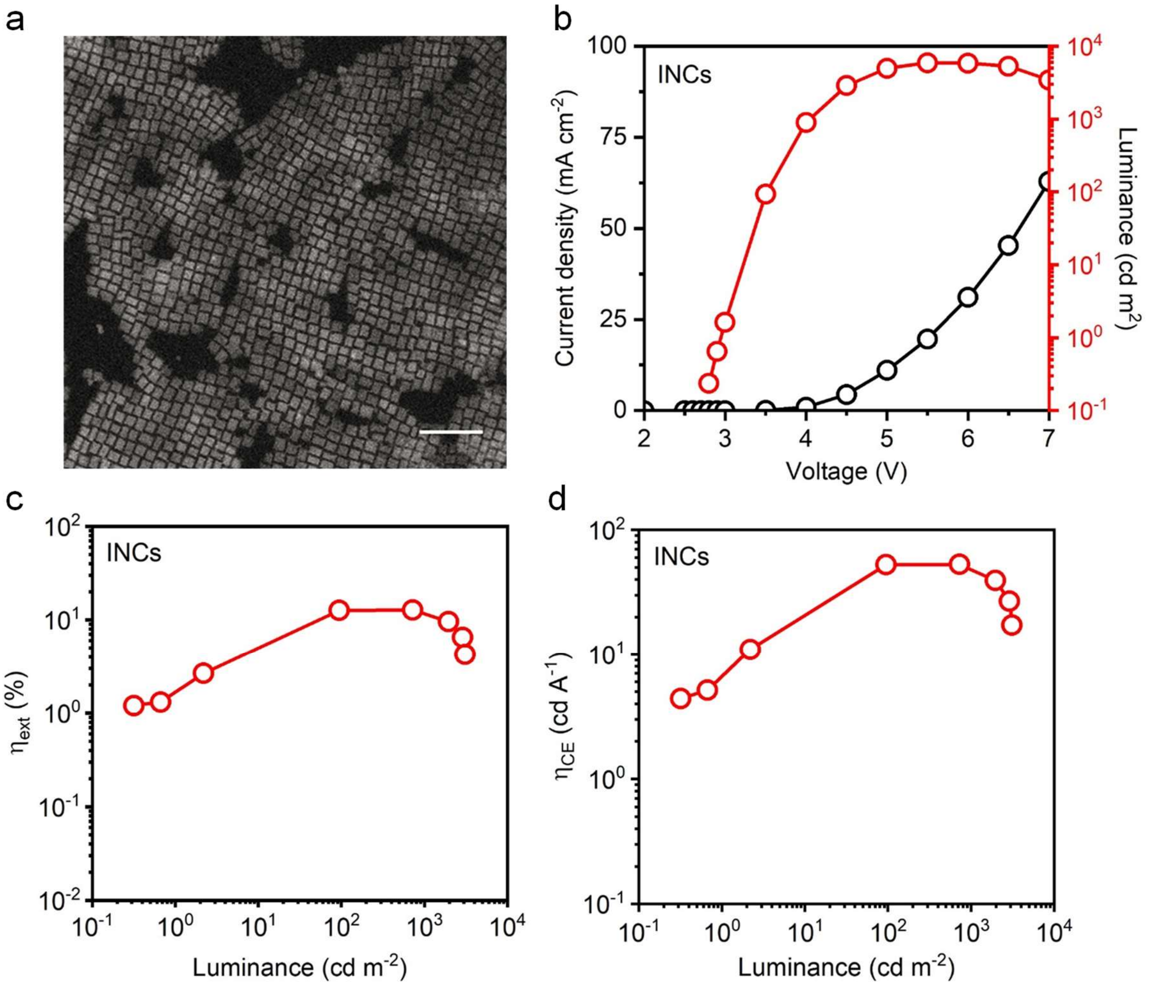

Supplementary Figure 38. Cryo-STEM image of isotropic LHP NCs (Scale bar: 50 nm). **a. (b-d)** EL characteristics of isotropic LHP NCs. Current density **b.** Luminance. **c.** as a function of driving voltage. **c.** η_{ext} as a function of luminance.

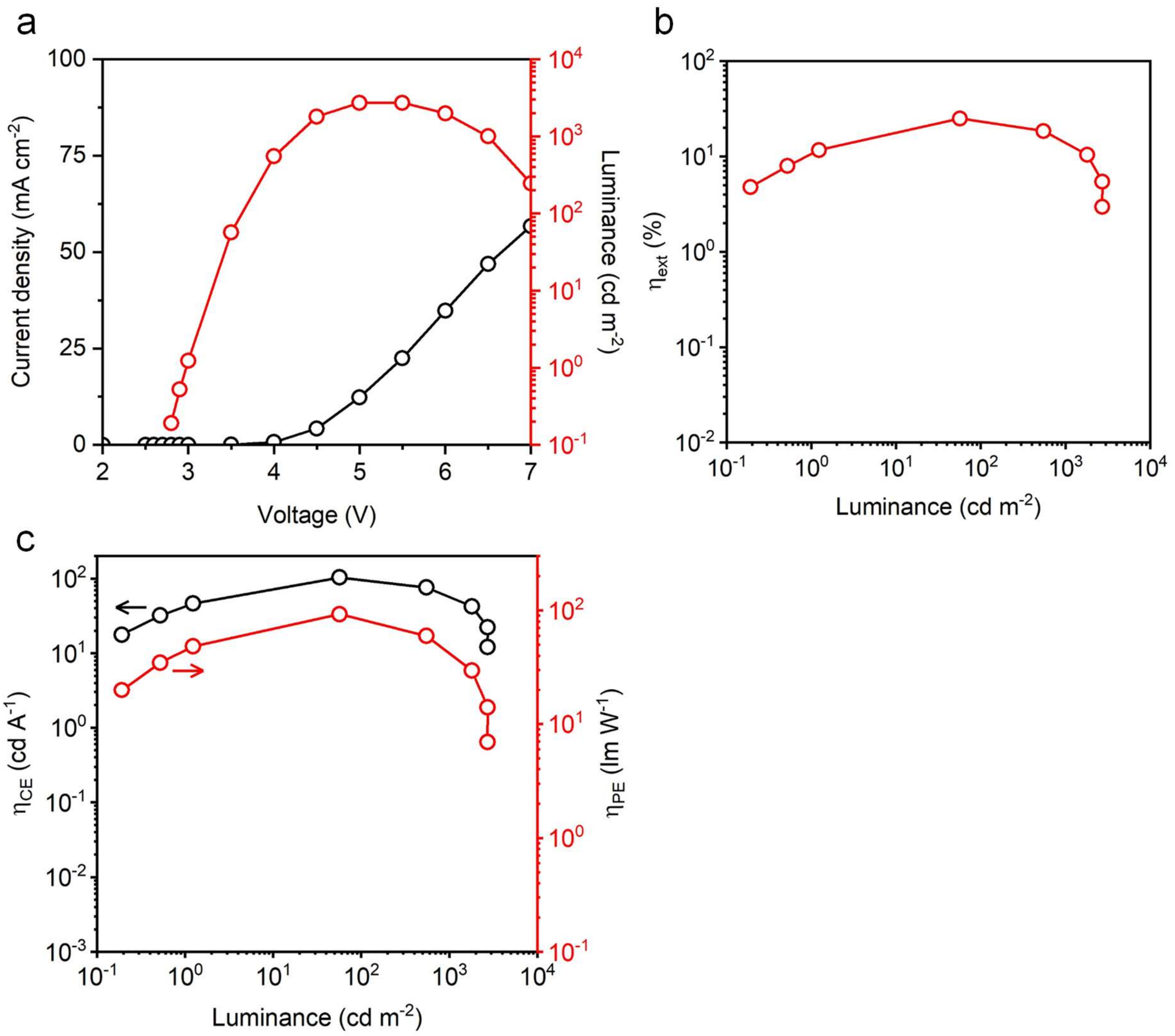

Supplementary Figure 39. EL characteristics of champion perovskite QD LED device. **a**, Current density and luminance as a function of voltage**. b.** η_{ext} as a function of luminance. **c**, η_{CE} and η_{PE} as a function of luminance.

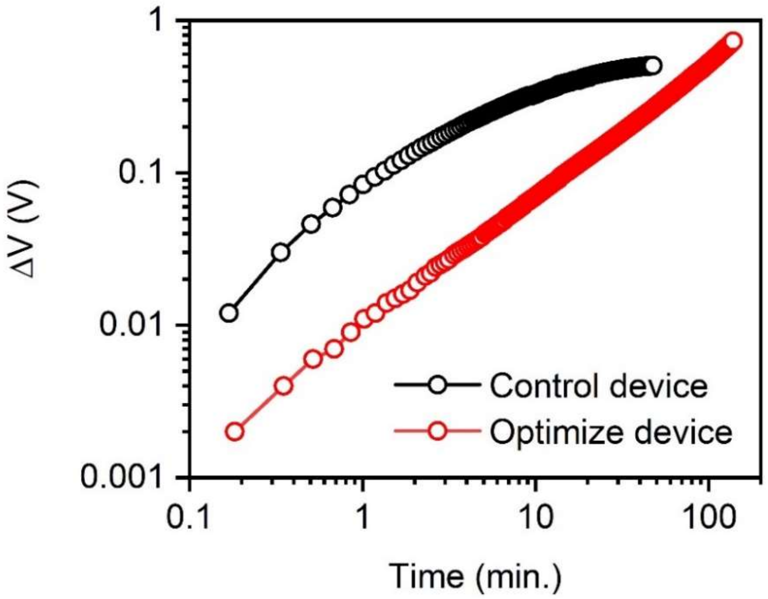

Supplementary Figure 40. Change in operating voltage (ΔV; offset to zero) in the control and optimal LED devices as a function of time under continuous electrical stress at a constant current density of 0.5 mA cm^{-2}.

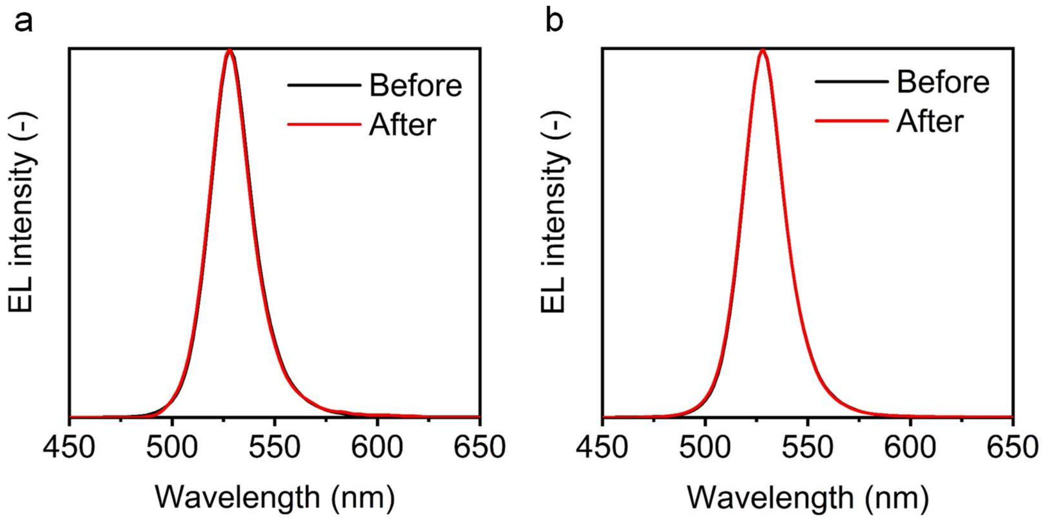

Supplementary Figure 41. EL spectra of **a.** control and **b.** optimize devices before and after operational lifetime test.

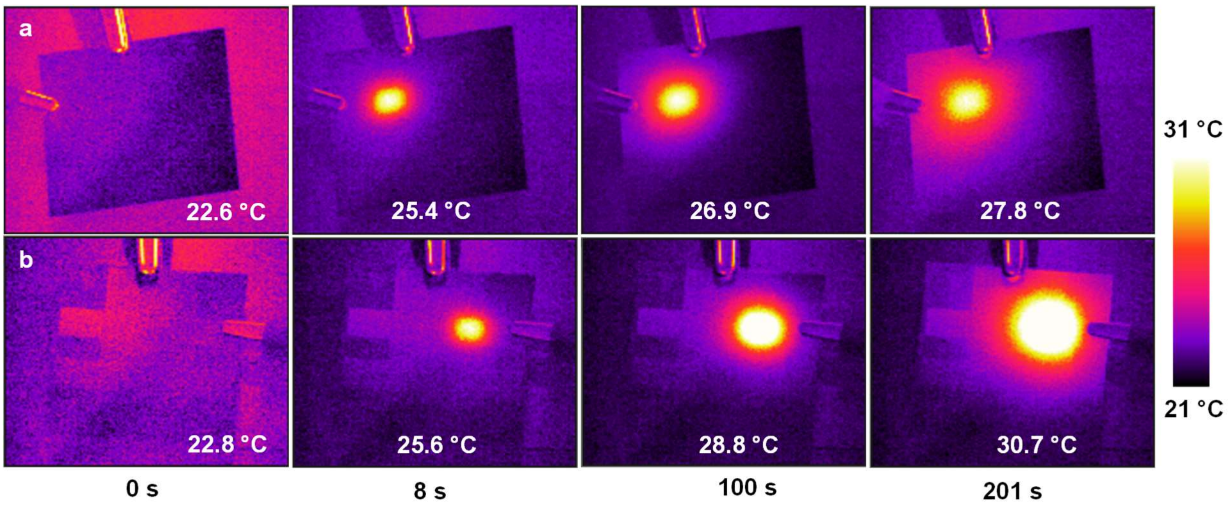

Supplementary Figure 42. The Joule heating effect during electrical operation of **a.** X-F6-TAPC and **b.** control devices characterized by an infrared camera.

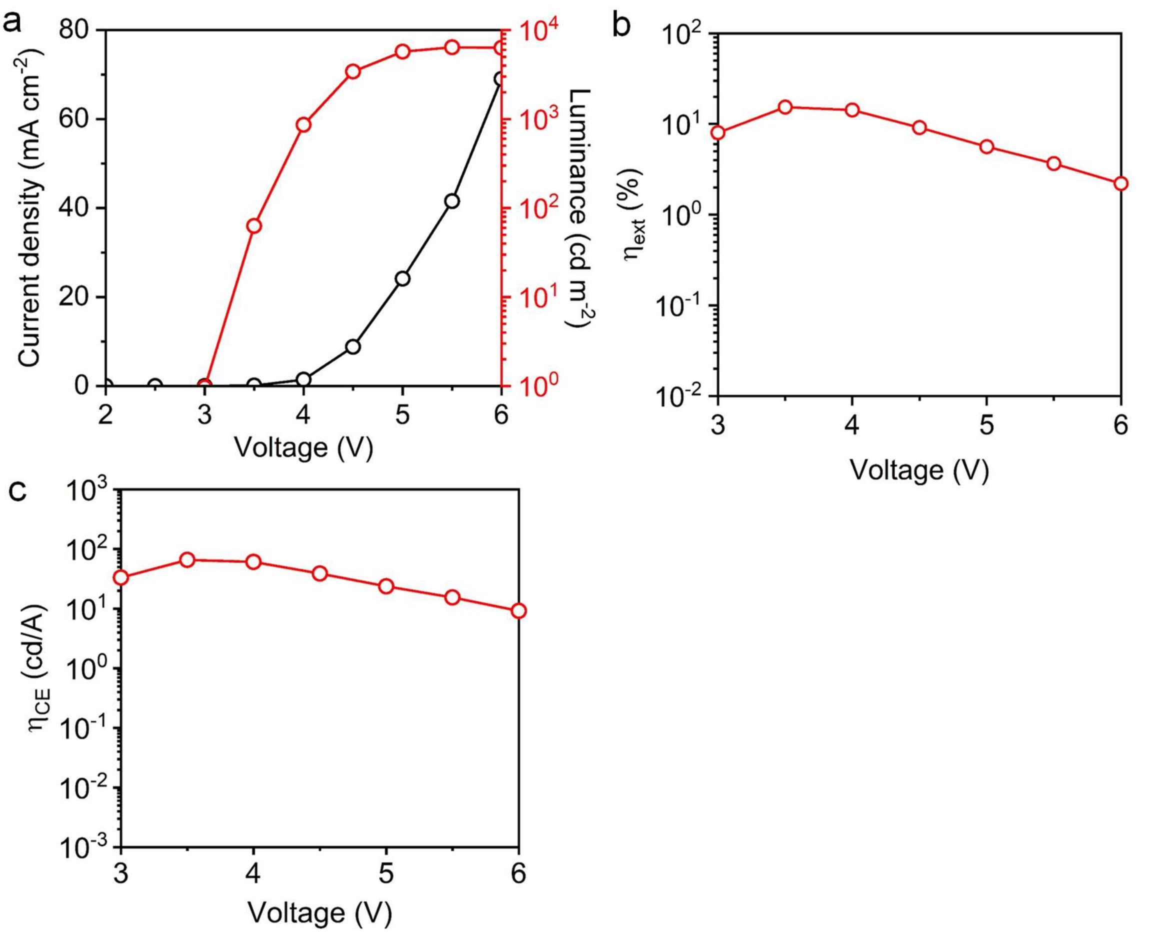

Supplementary Figure 43. EL characteristics of large area perovskite QD LED device. **a**, current density and luminance as a function of driving voltage. **b**, η_{ext} as a function of driving voltage. **c**, η_{CE} as a function of driving voltage.

Supplementary References

1. Tanaka K, Takahashi T, Ban T, Kondo T, Uchida K, Miura N. Comparative study on the excitons in lead-halide-based perovskite-type crystals $CH_3NH_3PbBr_3$ $CH_3NH_3PbI_3$. *Solid State Commun.* **127**, 619-623 (2003).

2. Grynberg G, Aspect A, Fabre C. *Introduction to Quantum Optics: From the Semi-classical Approach to Quantized Light*. Cambridge University Press (2010).

3. Becker MA*, et al.* Bright triplet excitons in caesium lead halide perovskites. *Nature* **553**, 189-193 (2018).

4. Peter Y. Yu MC. *Fundamental of Semiconductors*, 4th edn. Springer (2010).

5. Scott R*, et al.* Directed emission of CdSe nanoplatelets originating from strongly anisotropic 2D electronic structure. *Nat. Nanotechnol.* **12**, 1155-1160 (2017).

6. Markel VA. Introduction to the maxwell garnett approximation: tutorial. *J. Opt. Soc. Am. A* **33**, 1244-1256 (2016).

7. Hens Z, Moreels I. Light absorption by colloidal semiconductor quantum dots. *J. Mater. Chem.* **22**, 10406-10415 (2012).

8. Bohren CF, Huffman DR. *Absorption and Scattering of Light by Small Particles*, 1 edn. WILEY (1998).

9. Sihvola A. *Electromagnetic Mixing Formulas and Applications*, 1st edn. The Institution of Engineering and Technology (2008).

10. Galkowski K*, et al.* Determination of the exciton binding energy and effective masses for methylammonium and formamidinium lead tri-halide perovskite semiconductors. *Energy Environ. Sci.* **9**, 962-970 (2016).

11. Glaser T*, et al.* Infrared spectroscopic study of vibrational modes in methylammonium lead halide perovskites. *J. Phys. Chem. Lett* **6**, 2913-2918 (2015).

12. Sendner M*, et al.* Optical phonons in methylammonium lead halide perovskites and implications for charge transport. *Mater. Horiz.* **3**, 613-620 (2016).

13. Ruda HE, Shik A. Polarization-sensitive optical phenomena in semiconducting and metallic nanowires. *Phys. Rev. B* **72**, 115308 (2005).

14. Sattler KD. *Handbook of Nanophysics: Nanoelectronics and Nanophotonics*, 1 edn. CRC Press (2010).

15. Sercel PC, Lyons JL, Bernstein N, Efros AL. Quasicubic model for metal halide perovskite nanocrystals. *J. Chem. Phys.* **151**, 234106 (2019).

16. Kumar S, Jagielski J, Marcato T, Solari SF, Shih C-J. Understanding the ligand effects on photophysical, optical, and electroluminescent characteristics of hybrid lead halide perovskite nanocrystal solids. *J. Phys. Chem. Lett.* **10**, 7560-7567 (2019).

17. Shamsi J, Urban AS, Imran M, De Trizio L, Manna L. Metal halide perovskite nanocrystals: synthesis, post-synthesis modifications, and their optical properties. *Chem. Rev.* **119**, 3296-3348 (2019).

18. Fang H-H, Protesescu L, Balazs DM, Adjokatse S, Kovalenko MV, Loi MA. Exciton recombination in formamidinium lead triiodide: Nanocrystals versus thin films. *Small* **13**, 1700673 (2017).

19. Qin J, Liu X-K, Yin C, Gao F. Carrier dynamics and evaluation of lasing actions in halide perovskites. *Trends Chem.* **3**, 34-46 (2021).

20. Salehi A, Ho S, Chen Y, Peng C, Yersin H, So F. Highly efficient organic light-emitting diode using a low refractive index electron transport layer. *Adv. Opt. Mater.* **5**, 1700197 (2017).

21. Hassan Y*, et al.* Ligand-engineered bandgap stability in mixed-halide perovskite LEDs. *Nature* **591**, 72-77 (2021).

22. McDowall S, Butler T, Bain E, Scharnhorst K, Patrick D. Comprehensive analysis of escape-cone losses from luminescent waveguides. *Appl Opt* **52**, 1230-1239 (2013).

23. Mulder CL, Reusswig PD, Velázquez AM, Kim H, Rotschild C, Baldo MA. Dye alignment in luminescent solar concentrators: I. Vertical alignment for improved waveguide coupling. *Opt. Express* **18**, A79-A90 (2010).

24. Zannoni C. Order Parameters and Orientational Distributions in Liquid Crystals. In: *Polarized Spectroscopy of Ordered Systems* (eds Samori' B, Thulstrup EW). Springer Netherlands (1988).

25. Marcato T, Shih C-J. Molecular orientation effects in organic light-emitting diodes. *Helv. Chimi. Acta* **102**, e1900048 (2019).

26. Cho C*, et al.* The role of photon recycling in perovskite light-emitting diodes. *Nat. Commun.* **11**, 611 (2020).

27. Richter JM*, et al.* Enhancing photoluminescence yields in lead halide perovskites by photon recycling and light out-coupling. *Nat. Commun.* **7**, 13941 (2016).

28. Chance RR, Prock A, Silbey R. Molecular Fluorescence and Energy Transfer Near Interfaces. In: *Advances in Chemical Physics*) (1978).

29. Stranks SD, Hoye RLZ, Di D, Friend RH, Deschler F. The physics of light emission in halide perovskite devices. *Adv. Mater.* **31**, 1803336 (2019).

30. Luo X*, et al.* Mechanisms of triplet energy transfer across the inorganic nanocrystal/organic molecule interface. *Nat. Commun.* **11**, 28 (2020).

31. Hofmann A, Schmid M, Brütting W. The many facets of molecular orientation in organic optoelectronics. *Adv. Opt. Mater.* **n/a**, 2101004.

32. Yokoyama D. Molecular orientation in small-molecule organic light-emitting diodes. *J. Mater. Chem.* **21**, 19187-19202 (2011).

33. Frischeisen J, Yokoyama D, Adachi C, Brütting W. Determination of molecular dipole orientation in doped fluorescent organic thin films by photoluminescence measurements. *Appl. Phys. Lett.* **96**, 073302 (2010).

34. Front Matter. In: *Spectroscopic Ellipsometry*) (2007).

35. Yokoyama D, Sakaguchi A, Suzuki M, Adachi C. Horizontal orientation of linear-shaped organic molecules having bulky substituents in neat and doped vacuum-deposited amorphous films. *Org. Electron.* **10**, 127-137 (2009).

36. Wang F, Shan J, Islam MA, Herman IP, Bonn M, Heinz TF. Exciton polarizability in semiconductor nanocrystals. *Nat. Mater.* **5**, 861-864 (2006).

37. Scott R*, et al.* Time-resolved stark spectroscopy in CdSe nanoplatelets: Exciton binding energy, polarizability, and field-dependent radiative rates. *Nano Lett.* **16**, 6576-6583 (2016).

38. Leijtens T*, et al.* Modulating the electron–hole interaction in a hybrid lead halide perovskite with an electric field. *J. Am. Chem. Soc.* **137**, 15451-15459 (2015).

39. Röhr JA, Moia D, Haque SA, Kirchartz T, Nelson J. Exploring the validity and limitations of the Mott–Gurney law for charge-carrier mobility determination of semiconducting thin-films. *J. Phys. Condens. Matter* **30**, 105901 (2018).

40. Rizvi SMH, Mazhari B. An improved method for extraction of mobility from space charge limited current in organic semiconductor films. *J. Appl. Phys.* **121**, 155501 (2017).

41. Chen J, Ma P, Chen W, Xiao Z. Overcoming outcoupling limit in perovskite light-emitting diodes with enhanced photon recycling. *Nano Lett.* **21**, 8426-8432 (2021).

42. Daisaku T, Takashi T, Takayuki C, Soichi W, Junji K. Novel electron-transport material containing boron atom with a high triplet excited energy level. *Chem. Lett.* **36**, 262-263 (2007).

43. Liaptsis G, Meerholz K. Crosslinkable TAPC-based hole-transport materials for solution-processed organic light-emitting diodes with reduced efficiency roll-off. *Adv. Funct. Mater.* **23**, 359-365 (2013).

44. Shin H, Lee J-H, Moon C-K, Huh J-S, Sim B, Kim J-J. Sky-blue phosphorescent OLEDs with 34.1% external quantum efficiency using a low refractive index electron transporting layer. *Adv. Mater.* **28**, 4920-4925 (2016).

45. Jou J-H, Kumar S, Agrawal A, Li T-H, Sahoo S. Approaches for fabricating high efficiency organic light emitting diodes. *J. Mater. Chem. C* **3**, 2974-3002 (2015).

46. Umbach TE, Röllgen S, Schneider S, Klesper H, Umbach AM, Meerholz K. Low-refractive index layers in organic light-emitting diodes via electrospray deposition for enhanced outcoupling efficiencies. *Adv. Eng. Mater.* **22**, 1900897 (2020).

47. Kumar S*, et al.* Ultrapure green light-emitting diodes using two-dimensional formamidinium perovskites: Achieving recommendation 2020 color coordinates. *Nano Lett.* **17**, 5277-5284 (2017).

48. Fukagawa H, Oono T, Iwasaki Y, Hatakeyama T, Shimizu T. High-efficiency ultrapure green organic light-emitting diodes. *Mater. Chem. Front.* **2**, 704-709 (2018).

49. Liu Y*, et al.* Efficient blue light-emitting diodes based on quantum-confined bromide perovskite nanostructures. *Nat. Photon.* **13**, 760-764 (2019).
